# Sodium-Glucose Cotransport Protein 2 Inhibitors in Patients With Type 2 Diabetes and Acute Kidney Disease

**DOI:** 10.1001/jamanetworkopen.2023.50050

**Published:** 2024-01-03

**Authors:** Heng-Chih Pan, Jui-Yi Chen, Hsing-Yu Chen, Fang-Yu Yeh, Thomas Tao-Min Huang, Chiao-Yin Sun, Shiow-Ing Wang, James Cheng-Chung Wei, Vin-Cent Wu

**Affiliations:** 1 Graduate Institute of Clinical Medicine, College of Medicine, National Taiwan University, Taipei; 2 College of Medicine, Chang Gung University College of Medicine, Taoyuan, Taiwan; 3 Division of Nephrology, Department of Internal Medicine, Keelung Chang Gung Memorial Hospital, Keelung, Taiwan; 4 Community Medicine Research Center, Keelung Chang Gung Memorial Hospital, Keelung, Taiwan; 5 Division of Nephrology, Department of Internal Medicine, Chi Mei Medical Center, Tainan, Taiwan; 6 Department of Health and Nutrition, Chia Nan University of Pharmacy and Science, Tainan, Taiwan; 7 Graduate Institute of Clinical Medical Sciences, College of Medicine, Chang Gung University, Taoyuan, Taiwan; 8 Division of Chinese Internal Medicine, Center for Traditional Chinese Medicine, Chang Gung Memorial Hospital, Taoyuan, Taiwan; 9 School of Traditional Chinese Medicine, College of Medicine, Chang Gung University, Taoyuan, Taiwan; 10 Division of Nephrology, Department of Internal Medicine, National Taiwan University Hospital, Taipei, Taiwan; 11 National Taiwan University Hospital Study Group of Acute Renal Failure and Taiwan Consortium for Acute Kidney Injury and Renal Diseases, Taipei, Taiwan; 12 Institute of Medicine, Chung Shan Medical University, Taichung, Taiwan

## Abstract

**Importance:**

Sodium-glucose cotransport protein 2 inhibitors (SGLT-2is) have demonstrated associations with positive kidney-related and cardiovascular outcomes in patients with type 2 diabetes. However, the association of SGLT-2is with outcomes among patients with type 2 diabetes and acute kidney disease (AKD) remains unclear.

**Objective:**

To examine the long-term associations of SGLT-2is with mortality, major adverse kidney events (MAKEs), and major adverse cardiovascular events (MACEs) in patients with type 2 diabetes and AKD.

**Design, Setting, and Participants:**

This cohort study used global health care data (the TriNetX database) spanning from September 30, 2002, to September 30, 2022. Propensity score matching was used to select a cohort of patients, and follow-up was conducted with a maximum duration of 5 years (completed on September 30, 2022) or until the occurrence of an outcome or death.

**Intervention:**

The use of SGLT-2is.

**Main Outcomes and Measures:**

The primary outcomes measured were mortality, MAKEs, and MACEs. Adjusted hazard ratios (AHR) with 95% CIs were calculated to compare the risks between SGLT-2i users and nonusers, representing the mean treatment effect among the treated patients.

**Results:**

A total of 230 366 patients with AKD (mean [SD] age, 67.1 [16.4] years; 51.8% men and 48.2% women) were enrolled in the study, which had a median follow-up duration of 2.3 (IQR, 1.2-3.5) years. Among these, 5319 individuals (2.3%) were identified as SGLT-2i users. Among nonusers, the incidence of mortality was 18.7%, the incidence of MAKEs was 21.0%, and the incidence of MACEs was 25.8%. After propensity score matching, the absolute differences between SGLT-2i users and nonusers for incidence of mortality, MAKEs, and MACEs were 9.7%, 11.5%, and 12.3%, respectively. Based on the treated population, SGLT-2i use was associated with a significantly lower risk of mortality (AHR, 0.69 [95% CI, 0.62-0.77]), MAKEs (AHR, 0.62 [95% CI, 0.56-0.69]), and MACEs (AHR, 0.75 [95% CI, 0.65-0.88]) compared with nonuse. External validation using a multicenter cohort data set of 1233 patients with AKD patients who were SGLT-2i users confirmed the observed beneficial outcomes. Notably, the risk reduction associated with SGLT-2is remained significant even among patients without hypertension, those with advanced chronic kidney disease, and those not receiving other hypoglycemic agents.

**Conclusions and Relevance:**

In this cohort study of patients with type 2 diabetes and AKD, administration of SGLT-2is was associated with a significant reduction in all-cause mortality, MAKEs, and MACEs when compared with nonuse, underscoring the importance of SGLT-2is in care after acute kidney injury. These findings emphasize the potential benefits of SGLT-2is in managing AKD and mitigating the risks of major cardiovascular and kidney diseases.

## Key Points

**Question:**

Do sodium-glucose cotransport protein 2 inhibitors (SGLT-2is) have beneficial associations with mortality, major kidney events (MAKEs), and major adverse cardiovascular events (MACEs) in patients with type 2 diabetes and acute kidney disease (AKD)?

**Findings:**

In this cohort study of 230 366 patients, SGLT-2i use among those with type 2 diabetes and AKD was associated with significantly lower risks of mortality, MAKEs, and MACEs compared with nonuse.

**Meaning:**

These findings suggest that the use of SGLT-2is in patients with type 2 diabetes and AKD is associated with reduced mortality, MAKEs, and MACEs, highlighting their potential clinical implications.

## Teaser

This cohort study explores the associations of sodium-glucose cotransport protein 2 inhibitors (SGLT-2is) with morality and major adverse cardiovascular and kidney events among patients with type 2 diabetes and acute kidney disease.

## Introduction

Type 2 diabetes affects millions of people worldwide.^1,2,3^ It is an independent risk factor for acute kidney injury (AKI) and is associated with a decline in kidney function.^4^ Both type 2 diabetes and AKI are established risk factors for chronic kidney disease (CKD).^5,6^ Many patients with type 2 diabetes experience a decline in kidney function even before the onset of AKI, emphasizing their combined association with CKD development.^7^ Recently, acute kidney disease (AKD) has emerged as a transitional stage between AKI and CKD, lasting 7 to 90 days after an AKI episode.^8,9,10^ With the increasing incidence of AKI among hospitalized patients in various settings,^11^ AKD is also becoming increasingly prevalent. Su et al^12^ previously found that patients with AKD had a higher risk of all-cause mortality, end-stage kidney disease, incident CKD, and progressive CKD. Thus, effective management of AKD is vital to prevent further kidney damage and adverse outcomes.

Sodium-glucose cotransport protein 2 inhibitors (SGLT-2is) are a new class of oral hypoglycemic agents that have been shown to have beneficial associations with kidney-related and cardiovascular outcomes in various clinical settings, including type 2 diabetes, CKD, and heart failure.^13^ The primary mechanism of action of SGLT-2is is to inhibit the reabsorption of glucose and sodium in the kidneys, leading to a reduction in blood pressure, intraglomerular pressure, and albuminuria.^14^ Clinical trials^15,16,17,18^ have demonstrated that SGLT-2is are associated with a reduction in the risk of progression of CKD, improve kidney function, and reduce the risk of death in patients with type 2 diabetes. Several clinical trials^16,19^ have also reported that SGLT-2is might be associated with a lower risk of AKI in patients with type 2 diabetes. These findings may further support the protective benefits of SGLT-2is in preventing AKD in this patient population.

We conducted this study to explore the associations of SGLT-2is in a clinical setting of AKD among patients with type 2 diabetes. Using a longitudinal study design with a comprehensive global medical records database, we aimed to provide valuable evidence on the associations of SGLT-2is based on clinical data.

## Methods

The analysis of TriNetX data in this cohort study was approved by the institutional review board of Chi Mei Hospital, Tainan, Taiwan, and the institutional review boards of all participating hospitals. The TriNetX platform maintains compliance with the Health Insurance Portability and Accountability Act and General Data Protection Regulation, ensuring the utmost protection of patient information. The platform aggregates and consolidates only counts and statistical summaries of deidentified data from various institutions, without containing any individual-level data. Consequently, the Western Institutional Review Board has granted a waiver of informed consent for use of TriNetX data due to the absence of individual-level information.^20^ The current study was conducted in adherence to the principles outlined in the Declaration of Helsinki^21^ and followed the Strengthening the Reporting of Observational Studies in Epidemiology (STROBE) reporting guideline.

### Data Source, Study Protocol, and Patient Selection

All data used in this study were sourced from the TriNetX database, a global health collaborative clinical research platform. This platform has been used in numerous high-impact epidemiological studies.^20,22^ The data set used in this study consisted of a wide range of information, including patient demographics, diagnoses (based on *International Classification of Diseases, Tenth Revision, Clinical Modification* [*ICD-10-CM*] codes), procedures (based on *International Classification of Diseases, Tenth Revision, Procedure Coding System,* or *Current Procedural Terminology*), medications (coded according to the Veterans Affairs National Formulary), laboratory tests (based on LOINC [Logical Observation Identifiers Names and Codes]), genomics (coded according to the Human Genome Variation Society nomenclature), and records of use of health care services from 76 health care organizations, encompassing hospitals, primary care units, and specialists. This comprehensive data set included data from both insured and uninsured patients (eMethods in Supplement 1). For the purpose of this study, we constructed a cohort consisting of over 42 million participants, covering the period from September 30, 2002, to September 30, 2022.

### Prespecified Outcomes

The primary outcome was mortality, and the secondary outcomes were major adverse kidney events (MAKEs) and major adverse cardiac events (MACEs). MAKEs were defined as redialysis, dialysis dependence, or mortality, while MACEs were defined as cerebral infarction, hemorrhagic stroke, acute myocardial infarction, cardiogenic shock, or mortality.

### Cohort

**Figure 1.**
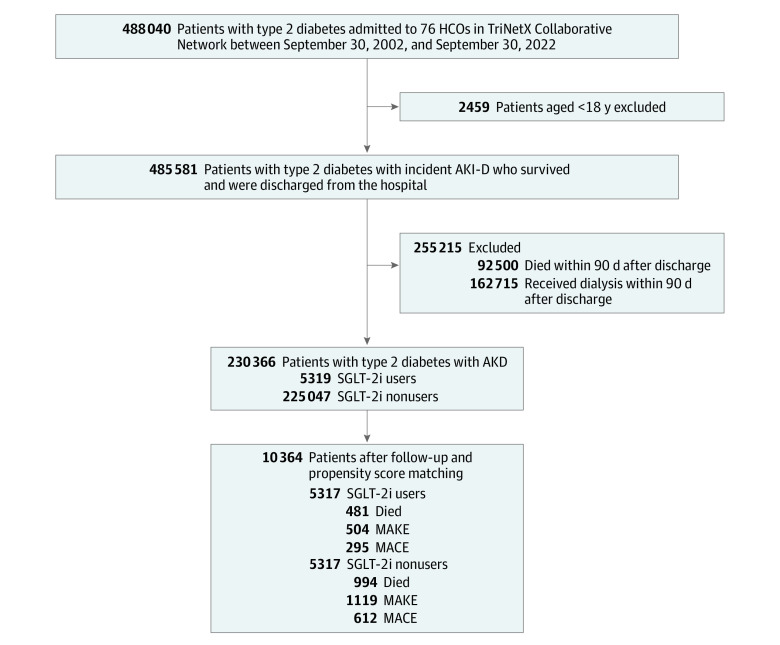
Patient Enrollment Algorithm AKI-D indicates dialysis-requiring acute kidney injury; HCO, health care organization; MACE, major adverse cardiac event; MAKE, major adverse kidney event; and SGLT-2i, sodium-glucose cotransport protein 2 inhibitor.

The objective of this study was to evaluate the associations with mortality rate within a cohort of 230 366 patients with type 2 diabetes and AKD who were admitted to targeted health care organizations (Figure 1). For all patients, the index date was defined as 90 days after hospital discharge, which originally marked the end of AKD. The inclusion criteria were (1) aged 18 to 90 years, (2) a diagnosis of type 2 diabetes, and (3) receipt of dialysis during hospitalization. Exclusion criteria were postdischarge redialysis or death within 3 months to minimize biases from acute, non–medication-related conditions and to ensure outcome attribution to SGLT-2is, enhancing data consistency.

We considered patients SGLT-2i users if they had received a prescription for an SGLT-2i during the study period. The cohort was subsequently divided into 2 groups: the SGLT-2i group (n = 5319) and the nonuser group (n = 225 047). Propensity score matching (PSM) was conducted on 25 characteristics at the index date, including age, sex, race and ethnicity (including American Indian or Alaska Native, Asian, Black, Native Hawaiian or Other Pacific Islander, White, and other or unknown), comorbidities, medications, and laboratory data. Data on race and ethnicity were considered potential covariates or adjustment variables in our study and were reported by the health care organizations that partner with TriNetX. The individuals in this cohort were longitudinally followed up to 5 years (to September 30, 2022) to estimate the risk of mortality. To mitigate protopathic or ascertainment bias, any events of primary and secondary outcomes that occurred before the index date were excluded and repeat PSM was performed.

### Covariates

To account for differences in baseline characteristics between the 2 groups, we extracted various covariates to provide insight into the study population. Demographic covariates, including age, sex, and race and ethnicity were incorporated, as well as comorbidities and concurrent medication use. Comorbidities were identified using *ICD-10-CM* codes. In addition, potential confounders such as physical examination results (eg, systolic blood pressure and body mass index) were considered. Laboratory tests analyzed in this study included white blood cell count, platelet count, estimated glomerular filtration rate (eGFR), proteinuria, and levels of total cholesterol, hemoglobin A_1c_, aspartate aminotransferase, sodium, potassium, and brain-type natriuretic peptide.

### Statistical Analysis

Variables were expressed either categorically (count and percentage) or numerically (mean [SD]), depending on the nature of the covariates. To account for potential confounding factors, we implemented PSM to create groups with comparable baseline characteristics, matching each SGLT-2i user to 1 nonuser. The built-in function of the TriNetX data set was used for this purpose, with greedy nearest neighbor matching based on factors such as age, sex, race and ethnicity, comorbidities, medication, and laboratory data. The balance of baseline characteristics was assessed using the standardized difference, with standardized difference less than 0.2 indicating a small difference.^23^ To reduce multicollinearity, continuous variables were preferred, and cases with missing data or who were lost to follow-up were excluded to ensure data completeness. The associations between the SGLT-2i user and control groups regarding primary and secondary outcomes were evaluated using the Cox proportional hazards regression model, from which adjusted hazard ratios (AHRs) were calculated.^24^ The dependence between users within matches was accounted for by using robust SEs. The assumption of proportional hazards was assessed using the generalized Schoenfeld approach integrated into the TriNetX platform, following the rigorous standards set by Grambsch and Therneau^25^ and Taquet et al.^26^ The follow-up period started after the index date, with a maximum duration of 5 years. Kaplan-Meier curves were used to estimate survival probabilities, considering 2-sided *P* < .05 as statistically significant. Risk analyses assessed the relative risks of adverse outcomes. Furthermore, subgroup analyses for mortality were conducted, focusing on variables including hypertension, advanced CKD, and medications, and interactions between SGLT-2is and covariates were thoroughly examined. To ensure the reliability of our findings, we conducted external validation using data from the Chang Gung Research Database^27^ and several sensitivity analyses, including eligible cases with different enrollment periods, and Cox proportional hazards regression with different covariates. Specificity analyses, positive outcome controls, and specified negative outcome controls were also performed (eMethods in Supplement 1). R software, version 3.2.2 (Free Software Foundation Inc), SAS software, version 9.2 (SAS Institute Inc), and Stata/MP software, version 16 (StataCorp LLC) were used for all analyses in this study, and statistics with a 2-sided *P* < .05 were considered significant.

## Results

### Study Population Characteristics

In this cohort of 230 366 patients with AKD, the mean (SD) age was 67.1 (16.4) years; 119 253 (51.8%) were men and 111 113 (48.2%) were women. We identified 5319 individuals who were SGLT-2i users and did not undergo dialysis or die within 3 months after discharge (Table 1). Therefore, the prevalence of SGLT-2i use was 2.3%. In addition, we identified 225 047 patients with type 2 diabetes who did not use SGLT-2is. The median follow-up duration for the entire cohort was 2.3 (IQR, 1.2-3.5) years, with the 90th percentile extending to 4.3 years. The presumptive causes of AKI are shown in eTable 1 in Supplement 1. In this study, sepsis was the most common cause of AKI (63.5%), followed by cardiorenal syndrome (38.9%). The eGFR and electrolyte levels after withdrawal of dialysis are shown in eTable 2 in Supplement 1.

**Table 1.** Baseline Characteristics of Patients Before and After Propensity Score Matching

Characteristic	Patient group					
	Before matching			After matching		
	SGLT-2i use (n = 5319)	Controls (n = 225 047)	Standardized difference	SGLT-2i use (n = 5317)	Controls (n = 5317)	Standardized difference
Demographic						
Age, mean (SD), y	63.8 (12.3)	67.4 (15.5)	0.25	63.8 (12.3)	64.2 (14.6)	0.03
Sex						
Men	3182 (59.8)	116 053 (51.6)	0.14	3181 (59.8)	3175 (59.7)	0.002
Women	2137 (40.2)	108 994 (48.4)	0.14	2136 (40.2)	2142 (40.3)	0.002
Race and ethnicity						
American Indian or Alaska Native	29 (0.5)	1009 (0.4)	0.01	26 (0.4)	30 (0.4)	0.01
Asian	141 (2.7)	4518 (2.0)	0.04	255 (4.8)	255 (4.8)	0.002
Black	1008 (19.0)	40 782 (18.1)	0.02	1122 (21.1)	1128 (21.2)	0.002
Native Hawaiian or Other Pacific Islander	14 (0.3)	369 (0.2)	0.02	70 (1.3)	54 (1.0)	0.03
White	3495 (65.7)	146 890 (65.3)	0.02	3493 (65.7)	3496 (65.8)	0.001
Other or unknown	632 (11.9)	26 513 (11.8)	0.003	710 (13.3)	704 (13.2)	0.001
Comorbidities						
Hyperlipidemia	3705 (69.7)	98 352 (43.7)	0.18	3703 (69.6)	3254 (61.2)	0.18
Chronic kidney disease	1806 (34.0)	63 527 (28.2)	0.11	3533 (66.4)	3454 (65.0)	0.01
Hyperuricemia	227 (4.3)	264 (0.1)	0.03	227 (4.3)	264 (5.0)	0.03
Congestive heart failure	2716 (51.1)	57 320 (25.5)	0.53	1806 (34.0)	1796 (33.8)	0.004
Ischemic heart diseases	2969 (55.8)	77 556 (34.5)	0.42	2967 (55.8)	2973 (55.9)	0.002
Cerebrovascular diseases	1205 (22.7)	45 417 (20.2)	0.05	1205 (22.7)	1187 (22.3)	0.01
Overweight	2617 (49.2)		0.42	2615 (49.2)	2582 (48.6)	0.01
COPD	992 (18.7)	34 211 (15.2)	0.08	991 (18.6)	961 (18.1)	0.02
Musculoskeletal disease	3699 (69.5)	123 816 (55.0)	0.28	3697 (69.5)	3704 (69.7)	0.003
Malignant neoplasm	138 (2.6)	5837 (2.6)	0.004	138 (2.6)	144 (2.7)	0.01
Medications						
Metformin	2906 (54.6)	54 087 (24.0)	0.65	2904 (54.6)	2890 (54.4)	0.01
Sulfonylureas	1209 (22.7)	27 458 (12.2)	0.27	1208 (22.7)	1205 (22.7)	0.001
DPP-4i	742 (13.9)	11 962 (5.3)	0.28	741 (13.9)	471 (8.9)	0.16
Acarbose	16 (0.3)	270 (0.1)	0.04	16 (0.3)	10 (0.2)	0.02
GLP-1 analogue	1024 (19.3)	5914 (2.6)	0.55	1024 (19.3)	346 (6.5)	0.39
Insulin	4719 (88.7)	146 114 (64.9)	0.56	4717 (88.7)	4813 (90.5)	0.06
Aspirin	3361 (63.2)	94 786 (42.1)	0.41	3359 (63.2)	3432 (64.5)	0.03
Clopidogrel	1167 (21.9)	26 360 (11.7)	0.27	1165 (21.9)	1119 (21.0)	0.02
Atorvastatin	3047 (57.3)	65 440 (29.1)	0.58	3045 (57.3)	3057 (57.5)	0.01
Allopurinol	416 (7.8)	12 105 (5.4)	0.09	416 (7.8)	386 (7.3)	0.02
Febuxostat	20 (0.4)	636 (0.3)	0.02	20 (0.4)	18 (0.3)	0.006
α-Blocker	886 (16.7)	26 354 (11.7)	0.13	886 (16.7)	780 (14.7)	0.06
β-Blocker	3889 (73.1)	112 360 (49.9)	0.47	3887 (73.1)	3685 (69.3)	0.08
CCB	2346 (44.1)	73 756 (32.8)	0.22	2345 (44.1)	2328 (43.8)	0.01
ACEI or ARB	4120 (77.5)	100 662 (44.7)	0.69	4118 (77.4)	3518 (66.2)	0.25
Laboratory						
BMI						
Mean (SD)	32.3 (7.1)	30.4 (7.3)	0.27	32.3 (7.1)	32.4 (7.3)	0.003
30 to 60	2034 (38.2)	53 344 (23.7)	0.31	2033 (38.2)	2014 (37.9)	0.01
25 to <30	1085 (20.4)	35 892 (15.9)	0.11	1084 (20.4)	1096 (20.6)	0.016
5 to <25	536 (10.1)	27 148 (12.1)	0.07	536 (10.1)	547 (10.3)	0.01
Missing or other	1664 (31.3)	108 663 (48.3)	0.35	1664 (31.3)	1660 (31.2)	0.002
WBC, mean (SD), ×10^3^/μL	9.5 (60.0)	11.5 (97.4)	0.02	9.5 (60.1)	11.9 (86.1)	0.03
Platelets, mean (SD), ×10^3^/μL	245.0 (102.4)	241.3 (112.5)	0.03	245.0 (102.4)	249.2 (115.8)	0.04
eGFR, mean (SD), mL/min/1.73m^2^	76.9 (32.9)	73.9 (40.8)	0.08	76.9 (32.9)	74.2 (40.5)	0.07
Proteinuria, mean (SD), mg/g	40.1 (36.1)	43.0 (37.9)	0.08	40.1 (36.1)	42.6 (39.5)	0.07
Total cholesterol level, mean (SD), mg/dL	155.4 (59.5)	160.7 (61.4)	0.09	155.4 (59.5)	158.2 (60.7)	0.05
HbA_1c_ level, %						
Mean (SD)	8.4 (2.3)	7.9 (2.5)	0.22	8.4 (2.3)	8.3 (2.2)	0.07
7.5 to 12.0	2507 (47.1)	47 556 (21.1)	0.56	2505 (47.1)	2482 (46.7)	0.01
6.5 to <7.5	1539 (28.9)	36 516 (16.2)	0.30	1538 (28.9)	1513 (28.5)	0.01
5.0 to <6.5	1080 (20.3)	48 357 (21.5)	0.04	1079 (20.3)	1030 (19.4)	0.02
Missing or other	193 (3.6)	92 618 (41.2)	1.01	195 (3.7)	292 (5.5)	0.08
AST level, mean (SD), U/L	31.7 (97.8)	42.5 (189.7)	0.07	31.7 (97.8)	47.7 (312.9)	0.07
Sodium level, mean (SD), mEq/L	137.4 (3.4)	137.2 (4.3)	0.07	137.4 (3.4)	137.0 (4.0)	0.11
Potassium level, mean (SD), mEq/L	4.1 (0.5)	4.0 (0.6)	0.19	4.1 (0.5)	4.0 (0.6)	0.12
BNP level, mean (SD), pg/mL	1006.7 (2877.3)	1111.7 (7702.5)	0.02	1006.7 (2877.3)	1443.8 (14 534.4)	0.04

Abbreviations: ACEI, angiotensin-converting enzyme inhibitor; ARB, angiotensin receptor blocker*;* AST, aspartate transaminase; BMI, body mass index (calculated as weight in kilograms divided by height in meters squared); BNP, brain-type natriuretic peptide; CCB, calcium channel blocker; COPD, chronic obstructive pulmonary disease; DPP-4i, dipeptidyl peptidase 4 inhibitor; eGFR, estimated glomerular filtration rate; GLP-1, glucagonlike peptide 1*;* HbA_1c_, hemoglobin A_1c_; SGLT-2i, sodium-glucose cotransport protein 2 inhibitor; WBC, white blood cell count.

SI conversion factors: To convert AST to μkat/L, multiply by 0.0167; BNP to ng/L, multiply by 1; cholesterol to mmol/L, multiply by 0.0259; platelets and WBC to 10^9^/L, multiply by 0.001; potassium to mmol/L, multiply by 1; sodium to nmol/L, multiply by 1.

Through PSM, we selected 5317 SGLT-2i users and 5317 matched nonusers (controls) for analysis. The mean (SD) age of the SGLT-2i group was 63.8 (12.3) years, which was lower than that in the control group (67.4 [15.5] years). The SGLT-2i group consisted of 3181 men (59.8%) and 2136 women (40.2%), and most patients were White (3493 [65.7%]). In the control group, 3175 (59.7%) were men and 2142 (40.3%) were women, and most were White (3496 [65.8%]). After PSM, the 2 groups had small and well-matched differences in age, sex, race and ethnicity, comorbidities, medication use, and most other laboratory results. The mean (SD) eGFRs in the SGLT-2i and control groups were 76.9 (32.9) and 74.2 (40.5) mL/min/1.73 m^2^, respectively. The SGLT-2i group had a greater proportion of angiotensin-converting enzyme inhibitor (ACEI) and angiotensin receptor blocker (ARB) users compared with the control group.

### Association of SGLT-2is With Main Outcomes Based on the Treated Population

After withdrawal from dialysis for AKI, the overall incidence rate of 5-year mortality was 13.9%; of MAKEs, 15.3%; and of MACEs, 21.0%. The 5-year all-cause mortality rate was 9.0% (481 of 5317) in the SGLT-2i group and 18.7% (994 of 5317) in the control group. Use of SGLT-2is was associated with a lower mortality rate (AHR, 0.69 [95% CI, 0.62-0.77]) (Table 2 and eTable 3 in Supplement 1). Additionally, a lower risk of MAKEs was observed in the SGLT-2i group (504 of 5317 [9.5%]) compared with the control group (1119 of 5317 [21.0%]; AHR, 0.62 [95% CI, 0.56-0.69]) (eTable 4 in Supplement 1).

**Table 2.** Incidence of Outcomes Among the SGLT-2i Users Compared With Controls After Prosperity Score Matching

Outcome	Patients with outcome, No./total No. (%)		AHR (95%CI)
	SGLT-2i group	Control group	
Primary outcome			
Mortality	481/5317 (9.0)	994/5317 (18.7)	0.69 (0.62-0.77)
Secondary outcome			
MAKE	504/5317 (9.5)	1119/5317 (21.0)	0.62 (0.56-0.69)
MACE	233/1732 (13.5)	690/2670 (25.8)	0.75 (0.65-0.88)

Abbreviations: AHR, adjusted hazard ratio; MACE, major adverse cardiac event; MAKE, major adverse kidney event; SGLT-2i, sodium-glucose cotransport protein 2 inhibitor.

**Figure 2.**
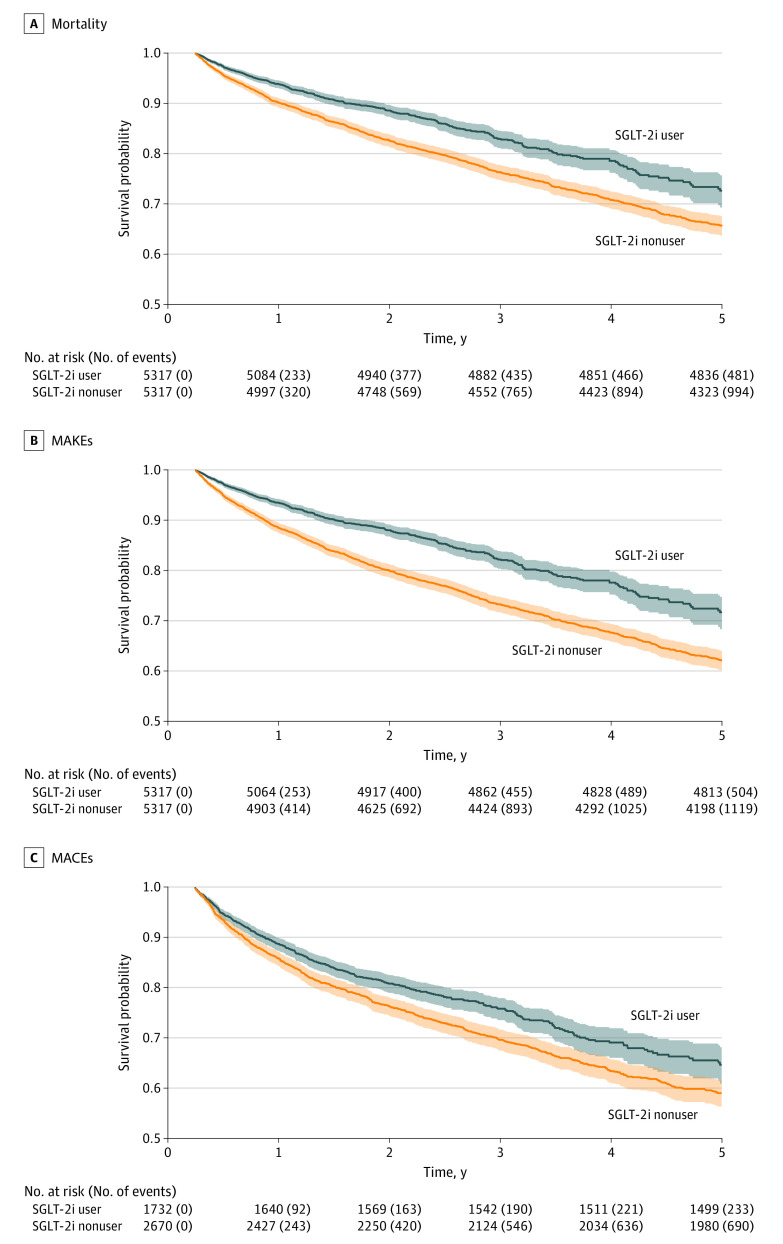
Kaplan-Meier Curves of Long-Term Outcomes of Interest All-cause mortality (A), major adverse kidney events (MAKEs) (B), major adverse cardiac events (MACEs) (C), and any initial outcome following the use of sodium-glucose cotransport protein 2 inhibitors (SGLT-2is) in a propensity score–matched counterpart are shown (log-rank *P* < .001 for all). Shaded areas indicate 95% CIs.

The baseline characteristics of the patients selected for MACEs analysis were comparable to those observed in the primary analysis (eTable 5 in Supplement 1). In the SGLT-2i group, a lower risk of MACEs was observed (233 of 1732 [13.5%]) compared with the control group (690 of 2670 [25.8%]; AHR, 0.75 [95% CI, 0.65-0.88]) (Table 2 and eTable 6 in Supplement 1). These results further support the effectiveness of SGLT-2is in reducing the occurrence of MACEs (Figure 2).

### Subgroup, Sensitivity, and Specificity Analyses Based on the Treated Population

**Figure 3.**
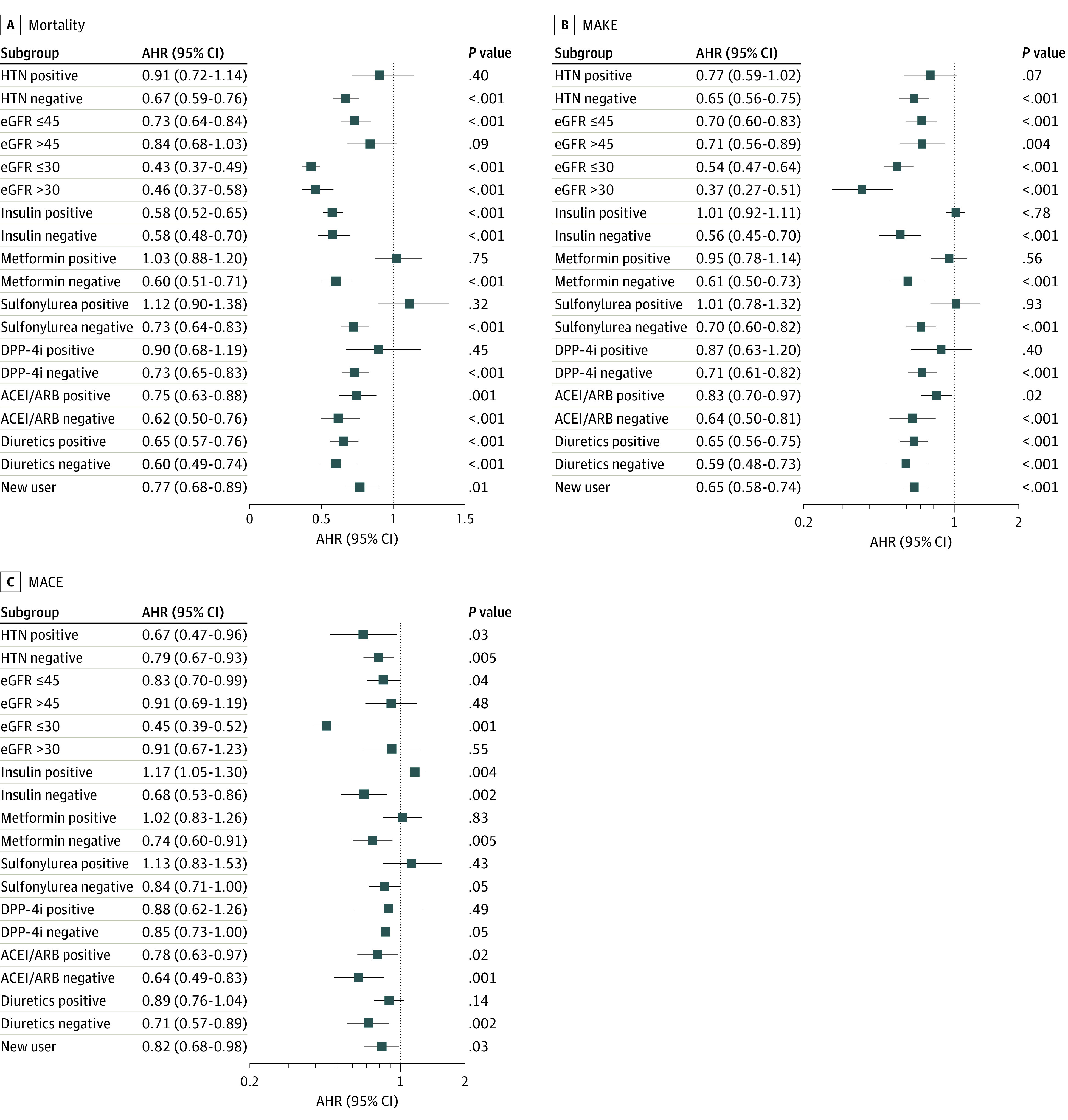
Subgroup Analysis Forest plots of adjusted hazard ratios (AHRs) for sodium-glucose cotransport protein 2 inhibitors (SGLT-2i) users vs nonusers during the acute kidney disease period regarding the long-term risks of sensitivity analysis for all-cause mortality, major adverse cardiac events (MACEs), and major adverse kidney events (MAKEs). The AHRS were adjusted for age, sex, and race and ethnicity due to their potential interactions with kidney disease. The vertical line indicates an AHR of 1.00; lower limits of 95% CIs with values greater than 1.00 indicate a significantly increased risk. ACEI indicates angiotensin-converting enzyme inhibitor; ARB, angiotensin receptor blocker; DPP-4i, dipeptidyl peptidase 4 inhibitor; eGFR, estimated glomerular filtration rate.

We conducted a subgroup analysis based on various comorbidities, including hypertension and advanced CKD, as well as medication use (Figure 3). The results suggest that the use of SGLT-2is was associated with a reduced risk of mortality, regardless of whether insulin or renin-angiotensin-aldosterone system (RAAS) blockers or diuretics were used. In addition, a significantly lower risk of mortality was observed in the SGLT-2i users who did not have hypertension (AOR, 0.67 [95% CI, 0.59-0.76]), had advanced CKD (eg, AOR for eGFR ≤45 mL/min/1.73 m^2^, 0.73 [95% CI, 0.64-0.84]), and were not taking other oral hypoglycemic agents (OHAs) (eg, AOR for metformin, 0.60 [95% CI, 0.51-0.71]).

The association between SGLT-2i use and a lower risk of MAKEs was observed consistently among patients with advanced CKD and among those who used RAAS blockers or diuretics. However, this association was more pronounced in the patients without hypertension and those who were not receiving insulin or other OHAs. Similarly, the association between a lower risk of MACEs and the use of SGLT-2i was observed consistently among patients with hypertension and those using RAAS blockers, and in particular among patients with advanced CKD and those who were not receiving insulin or other OHAs or diuretics.

Various models were used to assess the sensitivity analysis, incorporating eligible cases with varying selection criteria, as well as Cox proportional hazards regression models with diverse covariates (eTable 7 in Supplement 1). We also performed a sensitivity analysis to contrast the group of new users of SGLT-2is with those starting other second-line antihyperglycemic treatments, namely sulfonylureas, dipeptidyl peptidase 4 inhibitors, or pioglitazone (eTable 8 in Supplement 1). All sensitivity analyses yielded results that were in line with the primary approach.

Furthermore, in specific analyses of the outcome components, significant associations were observed between the use of SGLT-2is and a lower risk of several combinations of adverse events. These combinations included mortality and heart failure, mortality and myocardial infarction, mortality and stroke, and mortality and end-stage kidney disease (eFigure 1 in Supplement 1).

### Positive and Negative Outcome Controls

We then tested the occurrence of diabetic ketoacidosis and osteoporotic fractures as positive outcome controls to evaluate whether our approach would reproduce known associations. The risks of ketoacidosis (AHR, 1.36 [95% CI, 1.00-1.85]) and osteoporotic fractures (AHR, 1.39 [95% CI, 1.04-1.85]) were significantly higher in the SGLT-2i group (eFigure 1 in Supplement 1). To validate the robustness of our analytical approach, we also assessed various negative outcome controls, including atopic dermatitis, conjunctivitis, melanoma, lymphoma, and Hodgkin disease. These conditions were not anticipated to be associated with the use of SGLT-2is. Consistent with our expectations, the results revealed no associations between the use of SGLT-2is and any of the negative outcome controls when compared with the control group.

### External Validation

The results were further validated using 1233 patients with type 2 diabetes and AKD who were identified in the Chang Gung Research Database. Among this population, the prevalence of SGLT-2i use was 3.8% (47 of 1233). The SGLT-2i group was associated with a significantly lower risk of mortality (AHR, 0.43 [95% CI, 0.21-0.86]; *P* = .02), MAKEs (AHR, 0.39 [95% CI, 0.24-0.63]; *P* < .001), and MACEs (AHR, 0.47 [95% CI, 0.29-0.75]; *P* = .002) compared with the control group (eFigure 2 in Supplement 1).

## Discussion

In this cohort study, we compared patients with type 2 diabetes and AKD, including 5317 SGLT-2i users and 5317 matched controls (nonusers). According to our findings, 18.7% of patients with type 2 diabetes who did not use SGLT-2is experienced mortality after a median follow-up of 2.3 years. Those who used SGLT-2is were observed to have a significantly reduced risk of mortality. Furthermore, the risks of MAKEs and MACEs were also significantly lower in the SGLT-2i users. These results remained consistent and robust across various sensitivity analyses and were further validated using an external source. Moreover, the testing of both negative and positive outcome controls yielded expected results, further confirming the validity of our approach.

Our analysis of the risks and burdens of cardiovascular and kidney outcomes in a post-AKI care setting after acute dialysis revealed several significant findings. First, our findings have important implications for the management of AKD. Specifically, they highlight the importance of using SGLT-2is and implementing follow-up strategies for such patients. Second, our findings suggest that the use of SGLT-2is in patients with AKD and an eGFR of less than or equal to 30 or less than or equal to 45 mL/min/1.73 m^2^ and in those without hypertension may have significant implications for reducing the risks and burdens of major cardiovascular and kidney diseases. Therefore, it is crucial for clinicians to consider using SGLT-2is to address this growing public health concern.

### Associations of SGLT-2is With Survival

In this study, the use of SGLT-2is was associated with a significant survival advantage, even after accounting for important confounding factors. These findings are consistent with those of prior clinical trials.^16,19^ The risks in our study were consistent among the patients who did and did not receive concomitant ACEI or ARB therapy. After matching, a greater proportion of the SGLT-2i users received ACEIs or ARBs compared with the nonusers, which may be attributed to the proposed synergistic effect of SGLT-2is and RAAS blockers on lowering glucose levels in the management of patients with type 2 diabetes.^28^ In addition to lowering glycemic levels, SGLT-2is have been shown to have beneficial effects on metabolic markers, including decreases in blood pressure, body weight, and lipid profile.^29^ These effects may also explain their influence on enhancing survival.

Our analysis revealed that the benefits of SGLT-2is were most evident in patients without hypertension and without concomitant use of insulin or other OHAs. It is also possible that underlying factors or interactions are at play. Further randomized clinical trials are essential to validate these observations.

### Rationale for the Renoprotective and Cardioprotective Associations of SGLT-2is 

The findings of this study are consistent with accumulating evidence of the renoprotective and cardioprotective associations of SGLT-2i use in patients with type 2 diabetes.^30,31,32,33^ The mechanisms proposed to explain the renoprotective associations of SGLT-2is include enhancing the renal tubular glomerular feedback system and ameliorating metabolic dysfunction that may delay the development of diabetic kidney disease.^14,34^ The natriuretic, glycosuric, and osmotic diuretic effects of SGLT-2is have been shown to reduce cardiac preload and systemic congestion, consequently resulting in a cardioprotective effect.^35,36^ Our study provides evidence for the effectiveness of SGLT-2is in reducing the risk of redialysis and end-stage kidney disease, as well as decreasing cardiovascular events in patients with type 2 diabetes and AKD. Our findings underscore the potential of SGLT-2is as a versatile therapeutic approach for managing complications associated with diabetes.

### Clinical Implications

In the present study, we found that the prescription rate of SGLT-2is in patients with type 2 diabetes and AKD was extremely low (2.3%), despite recommendations by the American Diabetes Association.^37^ The guidelines advocate the use of SGLT-2is in patients with existing kidney disease. However, the low prescription rate in our study highlights the need to raise awareness and promote their use in managing diabetes complications. Despite considerable efforts to develop interventions for AKI, the clinical management of AKD remains challenging due to the persistent rise in the incidence of AKI^38^ and the limited number of available treatments.^39^ The promising role of SGLT-2is in improving the outcomes of patients with type 2 diabetes and AKD extends beyond glycemic control. Moreover, emerging evidence suggests that the renoprotective and cardioprotective associations of SGLT-2is are not limited to patients with type 2 diabetes, and they have been observed in patients without diabetes, those with CKD, and those with heart failure.^40,41,42,43,44,45,46^ Further investigations are needed to explore the potential benefits of SGLT-2is in patients with AKD in various clinical scenarios and settings.

### Limitations

There are several limitations in this study to consider. First, most of our participants were White, which may limit the generalizability of our results. We used global data from TriNetX to assess cardiovascular risks. Second, significant differences in comorbidities and medication use between the SGLT-2i users and nonusers may have introduced information bias. We applied 1:1 PSM and sensitivity analyses for confounders, with consistent results. Our standardized mean difference threshold of 0.2 for matching, less stringent than the usual 0.1, could cause group imbalances, which were assessed using subgroup and Cox proportional hazards regression analyses. Third, diseases were classified based on diagnostic codes, which may have led to underestimation of the presence of mild conditions or those occurring outside the medical system, and this may have led to ascertainment bias. Nevertheless, we attempted to minimize the influence of unknown confounders by analyzing medication use as a proxy. Fourth, despite our rigorous approach, there is a possibility of misclassification bias and residual confounding in our study. Unmeasured or unknown characteristics could still be associated with the risk of adverse outcomes, introducing potential confounding factors. To address selection bias, we conducted a specificity test comparing unrelated events between SGLT-2i users and nonusers, which showed no significant difference, indicating that other sources of recall bias were unlikely to be significant. Fifth, TriNetX’s tools limited our use of competing risks models, potentially biasing results if deceased patients had higher risks for other outcomes. We included mortality in MAKE and MACE outcomes, considering the higher death risk after post-AKI dialysis weaning. Sixth, our study’s retrospective design and lack of raw data hindered a time-varying analysis. We chose an intention-to-treat approach, providing insights within our data and design constraints. Seventh, although we used validated outcome definitions and PSM to minimize bias, misclassification bias and residual confounding may still be present due to the inherent limitations of using a health care database for an electronic health records study. However, our results were further validated using external data from a tertiary medical center outside the TriNetX system, and the conclusions were the same, providing additional support for the robustness of our findings. Eighth, our SGLT-2i analysis included both established and new users to provide a comprehensive view. We conducted a new-user design sensitivity analysis to address potential biases from concurrent medication use, such as sulfonylureas, dipeptidyl peptidase 4 inhibitors, or pioglitazone, ensuring a robust evaluation of our results. Finally, we excluded cases with incomplete outcome data to preserve data integrity, which may introduce selection bias. Tenth, our data set lacked detailed causes for redialysis or death, limiting our understanding of these outcomes.

## Conclusions

In this cohort study, we provide compelling clinical evidence supporting the associations of SGLT-2is in reducing the risk of mortality among patients with type 2 diabetes and AKD during a median follow-up period of 2.3 years. Use of SGLT-2is was associated with a lower risk of MAKEs and MACEs compared with nonuse. These findings highlight the potential benefit of SGLT-2is and suggest that clinicians should consider incorporating them into the management of type 2 diabetes with AKD.

## Notes

Supplement 1. **eMethods.** TriNetX Database, Cohorts, and Definitions **eTable 1.** Presumptive Causes of AKI **eTable 2.** Kidney Function and Electrolytes After Withdrawal of Dialysis **eTable 3.** Risk of Mortality in Patients With Type 2 Diabetes and AKD: Comparison Between SGLT-2I Users and Nonusers After Propensity Score Matching **eTable 4.** Risk of MAKE in Patients With Type 2 Diabetes and AKD: Comparison Between SGLT-2I Users and Nonusers After Propensity Score Matching **eTable 5.** Comparing SGLT-2I Users and Nonusers in Relation to MACE **eTable 6.** Risk of MACE in Patients With Type 2 Diabetes and AKD: Comparison Between SGLT-2I Users and Nonusers After Propensity Score Matching **eTable 7.** Sensitivity Analysis for All-Cause Mortality Between SLGT-2I Users and Nonusers **eTable 8.** Sensitivity Analysis for All-Cause Mortality, MAKE, and MACE Between SLGT-2I Users and Other Active Treatment (Sulfonylureas, Dipeptidyl Peptidase-4 Inhibitor, or Pioglitazone) Users in a New-User Design **eFigure 1.** Positive Outcome Control, Negative Outcome Control, and Specificity Analysis **eFigure 2.** External Validation by CGRD Database
eReferences

Supplement 2.
Data Sharing Statement
