# Supplementary material for: Sodium-Glucose Cotransport Protein 2 Inhibitors in Patients With Type 2 Diabetes and Acute Kidney Disease
Source: JAMA Netw Open. 2024 Jan 3;7(1):e2350050. doi: 10.1001/jamanetworkopen.2023.50050 (PMC10765268; doi:10.1001/jamanetworkopen.2023.50050)
Supplement: Supplement 1. — eMethods. TriNetX Database, Cohorts, and Definitions eTable 1. Presumptive Causes of AKI eTable 2. Kidney Function and Electrolytes After Withdrawal of Dialysis eTable 3. Risk of Mortality in Patients With Type 2 Diabetes and AKD: Comparison Between SGLT-2I Users and Nonusers After Propensity Score Matching eTable 4. Risk of MAKE in Patients With Type 2 Diabetes and AKD: Comparison Between SGLT-2I Users and Nonusers After Propensity Score Matching eTable 5. Comparing SGLT-2I Users and Nonusers in Relation to MACE eTable 6. Risk of MACE in Patients With Type 2 Diabetes and AKD: Comparison Between SGLT-2I Users and Nonusers After Propensity Score Matching eTable 7. Sensitivity Analysis for All-Cause Mortality Between SLGT-2I Users and Nonusers eTable 8. Sensitivity Analysis for All-Cause Mortality, MAKE, and MACE Between SLGT-2I Users and Other Active Treatment (Sulfonylureas, Dipeptidyl Peptidase-4 Inhibitor, or Pioglitazone) Users in a New-User Design eFigure 1. Positive Outcome Control, Negative Outcome Control, and Specificity Analysis eFigure 2. External Validation by CGRD Database eReferences [file jamanetwopen-e2350050-s001.pdf]

Supplementary Online Content

Pan HC, Chen JY, Chen HY, et al. Sodium-glucose cotransport protein 2 inhibitors in patients with type 2 diabetes and acute kidney disease. JAMA *Netw Open*. 2024;6(1):e2350050. doi:10.1001/jamanetworkopen.2023.5005

eMethods. TriNetX Database, Cohorts, and Definitions
eTable 1. Presumptive Causes of AKI
eTable 2. Kidney Function and Electrolytes After Withdrawal of Dialysis
eTable 3. Risk of Mortality in Patients With Type 2 Diabetes and AKD: Comparison Between SGLT-2I Users and Nonusers After Propensity Score Matching
eTable 4. Risk of MAKE in Patients With Type 2 Diabetes and AKD: Comparison Between SGLT-2I Users and Nonusers After Propensity Score Matching
eTable 5. Comparing SGLT-2I Users and Nonusers in Relation to MACE
eTable 6. Risk of MACE in Patients With Type 2 Diabetes and AKD: Comparison Between SGLT-2I Users and Nonusers After Propensity Score Matching
eTable 7. Sensitivity Analysis for All-Cause Mortality Between SLGT-2I Users and Nonusers
eTable 8. Sensitivity Analysis for All-Cause Mortality, MAKE, and MACE Between SLGT-2I Users and Other Active Treatment (Sulfonylureas, Dipeptidyl Peptidase-4 Inhibitor, or Pioglitazone) Users in a New-User Design
eFigure 1. Positive Outcome Control, Negative Outcome Control, and Specificity Analysis
eFigure 2. External Validation by CGRD Database
eReferences

This supplementary material has been provided by the authors to give readers additional information about their work.

©2024 Pan HC et al. *JAMA Network Open*.

eMethods. TriNetX Database, Cohorts, and Definitions

Introduction to the TriNetX database

1. TRINETX FEDERATED DATA NETWORK

TriNetX was initially developed with the aim of making collaborative industry–academia clinical trial research more efficient. It enables researchers to use real-world data to design trials that have the potential to meet their accrual requirements, and to identify performance sites that should be invited to open a trial. Beginning in 2015, TriNetX contacted health care organizations (HCOs) that had established i2b2 research repositories to join the network as data providers. Over the years, the data harmonization processes within TriNetX have evolved and improved, removing the requirement for data sources to have an i2b2 repository. TriNetX functions on a hub-and-spoke model, wherein Pharma and CRO sponsors pay a subscription fee to query for aggregate counts from the HCOs in the network, which are populated with deidentified patient data. This business model has proven successful, with 14 leading Pharma and CRO sponsors subscribed, and 76 HCO data providers in the network.(1)

2. NETWORK INFRASTRUCTURE

TriNetX is a multitenant software-as-a-service platform that utilizes Amazon Web Services (AWS) for its architecture, as depicted in Figure 1. Health Care Organizations (HCO) data accessible through the TriNetX network is stored on the appliance that is located at each HCO data center. During the onboarding process, the data is loaded onto the appliance with an extract-transform-load process that leverages the existing capabilities and scripting of the TriNetX agent. In addition to i2b2, TriNetX supports the loading of data from other source systems with a combination of its product and service capabilities.(1)

3. SECURITY

TriNetX is deployed on a secure Health Insurance Portability and Accountability Act-compliant (HIPAA) virtual private cloud hosted by AWS. This cloud meets Federal Risk and Authorization Management Program (FedRAMP), NIST 800-53, and other industry-standard security certifications. Access to TriNetX is secured with Transport Layer Security (TLS) and a 2048-bit security certificate. Services hosted behind AWS's Elastic Load Balancer are configured using the AWS Elastic Load Balancer Security Policy 2015-05. (https://aws.amazon.com/security/)

The TriNetX appliance is highly secure and locked down with no extraneous processes running. All communication is initiated outbound, and penetration and vulnerability tests are regularly conducted against the hosted application environment. Expert attestation of the appliance's security is available to TriNetX members, with full documentation available.(1) TriNetX is the global federated health

©2024 Pan HC et al. *JAMA Network Open*.

research network providing access to electronic medical records (diagnoses, procedures, medications, laboratory values, genomic information) across large healthcare organizations (HCOs). This report was run on the set of HCOs grouped into a network called Research. This network included 76 HCO(s).

4. CLINICAL DATA, CONTROLLED TERMINOLOGIES, AND SEMANTIC MAPPING

TriNetX is an innovative healthcare network that provides demographic, diagnosis, procedure, medication, and laboratory data for research purposes. In addition, TriNetX has recently expanded their offering to include tumor registry and molecular genomic data. Furthermore, they plan to add vital signs and other observation data relevant to oncology and pulmonology in the near future. This will provide researchers with a comprehensive and real-world dataset of clinical data to facilitate evidence-based research. The data ingested by the TriNetX appliance varies in origin depending on the healthcare organization (HCO). Some HCOs extract data directly from their electronic health records (EHRs), while others have data warehouses with varying common data models, (2) such as i2b2 and observational health data sciences and informatics. A typical commercial EHR uses a myriad of proprietary code system standards or terminology standards that may vary by country, such as the United States' Clinical Modification version of the International Classification of Diseases (ICD), Tenth Revision. In the United States, procedures are coded using ICD-10-PCS and Current Procedural Terminology (CPT), yet there is no accepted standard for procedures in other countries. Many EHRs incorporate proprietary drug data, including First DataBank, Wolters Kluwer’s Medi-Span, and Cerner’s Multum, each of which has a different identifier for the same drug. Medications may also be coded to national drug codes or to anatomic therapeutic chemical codes, used in many European countries, or local codes. Additionally, laboratory information systems at HCOs and commercial laboratories rarely use standard codes, such as Logical Observation Identifiers Names and Codes (LOINCs), for test results.

5. DATA QUALITY

Data quality is a major challenge when it comes to the proper use of research data, and can potentially compromise the validity of research results.(3) Although the adoption of Electronic Health Records (EHRs) has grown exponentially due to federal incentives and meaningful use requirements, the quality of the data within them, and therefore the data used for research, is still improving. As EHRs are primarily designed for billing and patient care functions, the data may not be of the highest quality for research purposes.16 To address this issue, a comprehensive data-quality framework and approach are needed.(4) Research on data quality is limited and generally focuses on assessing the quality of data in a single system or institution, (5)as well as determining if the data is good enough for its primary purpose, which is providing clinical care to patients. TriNetX has developed a comprehensive methodology to assess the quality of the data it uses. This methodology,

which consists of four Cs—cleanliness, consistency, correctness, and completeness—takes into account the data extracted from the source systems, which is then transformed, cleaned-up, deduplicated, de-identified, optionally obfuscated, and semantically mapped.

6. ANALYSIS SPECIFICATIONS

The Compare Outcomes Analytic offers four different types of analyses: Measure of Association, Survival, Number of Instances, and Lab Result Distribution. The first three analyses have the option to "exclude patients with outcomes prior to the window." This option is useful when analyzing outcomes that are chronic diseases, as patients who have already developed the outcome are not at risk of developing it during the time window. When the "exclude patients with outcomes prior to the time window" option is unchecked, all patients in the cohort will be included in the analysis, regardless of whether they had the outcome prior to the time window. However, if this option is checked, patients will be excluded if their medical record indicates that they had the outcome before the start of the time window. This exclusion will apply to all patients who had the outcome prior to the index event, and any patients who develop the outcome between the index event and the start of the time window will also be excluded if the time window starts some days after the index event.

7. MEASURE OF ASSOCIATION ANALYSIS

The Measure of Association Analysis assesses the fraction of patients with a specified outcome. The output summary includes the number of patients in each cohort meeting the query criteria, the number of patients with the outcome in each cohort, and the risk of the outcome in each cohort. We made adjustments for various factors including demographics (age, sex, race), health conditions (underlying chronic kidney disease, hyperuricemia, congestive heart failure, ischemic heart diseases, cerebrovascular diseases, overweight, chronic obstructive pulmonary disease, musculoskeletal disease, malignancy), medications (Metformin, Sulfonylureas, Acarbose, Insulin, Aspirin, Clopidogrel, Atrovastatin, Allopurinol, Febuxostat, alpha-blocker, beta-blocker, calcium channel blocker), and clinical metrics (body mass index, leukocyte count, platelet count, estimated glomerular rate, proteinuria level, total cholesterol, glycohemoglobin, aspartate transaminase, B-type natriuretic peptide). Furthermore, the output includes the Risk Difference (the difference in the risks in cohorts of type 2 diabetes with AKD who received and not received SGLT-2 inhibitors), Risk Ratio (the ratio of the risks in SGLT-2i users cohort and non-user cohort), and Odds Ratio (the ratio of the odds in SGLT-2i users cohort and non-user cohort).

8. SURVIVAL ANALYSIS

The Kaplan-Meier Analysis estimates the probability of the outcome at a respective time interval (in this analysis, daily time intervals are used). To account for patients who exited the cohort during the analysis period, censoring is applied, whereby these patients are removed from the

©2024 Pan HC et al. *JAMA Network Open*.

analysis after the last fact in their record. The output summary includes the number of patients in each cohort (meeting the query criteria), the number of patients with the outcome in the time window, median survival (the number of days when survival drops below 50%; the "-" indicates survival does not drop below 50% during the time window), and survival probability at the end of the window (the % survival at the end of the window). Furthermore, the Log-Rank Test, Hazard Ratio, and test for Proportionality are employed.

9. NUMBER OF INSTANCES ANALYSIS

The Number of Instances Analysis is a method that calculates the frequency of an outcome within a given time window. This analysis includes two settings: patients with zero instances and the definition of an instance. When selecting to exclude patients with zero instances, these patients are not included in the calculations for mean number of instances, standard deviation, or median. The histogram that displays the distribution of patients by number of instances will not include a bar for zero. Alternatively, by selecting to include patients with zero instances, the mean, standard deviation, and median for the number of instances will reflect the entire patient population, including those with zero instances. The histogram will include a bar for zero instances. The definition of an instance impacts how the counts are analyzed. When selecting Date, each calendar date on which any of the terms selected in the outcome are recorded will represent one instance. For instance, if the outcome is "Med A or Med B," and a patient has "Med A" on January 3, both medications on January 4, and "Med B" on January 6, then that patient is considered to have three instances, representing January 3, January 4, and January 6. It is important to note that if an outcome occurs across multiple dates, only the start date is tracked for the purpose of counting instances. For instance, a patient who begins a hospital stay on January 1, ends on January 3, begins another stay on January 10, and ends on January 15, is considered to have two instances of the outcome. Selecting Visit as an instance will count any visit that includes the outcome as one instance, regardless of how many times it occurred. For example, a patient administered an analgesic on each of the three days of an inpatient stay following some index event. If analgesic is an outcome, these three administrations will represent only one instance because they are associated with the same visit. The output summary includes the count of patients in the cohort, the count of patients in the cohort that had the outcome in the time window, the mean, standard deviation, and median of the counts, and the median (1+ instances) when patients with zero instances are included in the analysis. Additionally, T-Test statistics testing for the difference between the cohorts are included.

10. LABORATORY RESULTS ANALYSIS

In the analysis, only lab results that are relevant to the outcomes are included. Furthermore, only the most recent lab values within the time window are taken into account. For numeric lab results, the outcome summary contains the number of patients in the cohort who meet the query criteria, the

number of patients with the outcome within the time window, the mean, and the standard deviation of the lab values in the cohort. Additionally, T-Test statistics are provided to evaluate the difference between the cohorts. For non-numeric lab results, the counts of Negatives, Positives, and Unknowns are reported, and these percentages of the total counts are represented in the form of a bar chart.

11. LIMITATION

The TriNetX platform operates on individual-level data. However, as researchers, we do not have direct access to this individual data. Instead, the platform provides us with aggregated counts and statistical summaries of de-identified data, ensuring the privacy and confidentiality of the individuals represented in the data. The TriNetX platform pools data from various participating institutions, and while it processes individual-level data, it only allows researchers like us to interact with and analyze the aggregated and summarized data. This ensures compliance with both the Health Insurance Portability and Accountability Act and the General Data Protection Regulation.

©2024 Pan HC et al. *JAMA Network Open*.

External validation

We utilized data from the Chang Gung Research Database (CGRD), which is the most extensive collection of electronic medical records (EMR) across multiple institutions in Taiwan(6). This database provides researchers with convenient access to standardized patient-level data, enabling efficient utilization for a range of studies related to SGLT2 inhibitors. These studies encompass investigations into renoprotective and cardioprotective of SGLT2 inhibitors, as well as the assessment of their adverse side effects(7, 8, 9, 10, 11). The CGRD is a comprehensive collection of daily medical records gathered prospectively from seven branches of Chang Gung Memorial Hospital in Taiwan since January 2001. This database encompasses a significant volume of medical information, with an average of 500,000 emergency department visits, 8,500,000 outpatient visits, and over 280,000 admissions to 10,070 beds annually(6). The CGRD includes detailed personal information about patients, such as gender, body weight, height, lifestyle, and birth date. It also contains laboratory findings, pathology reports, imaging exam results, and comprehensive information about every emergency, inpatient, and outpatient visit. The database uses International Classification of Diseases, 9th and 10th revision, Clinical Modification (ICD-9-CM and ICD-10-CM) codes for classifying underlying diseases, reasons for admission, and details of emergency and outpatient visits. To prioritize patient privacy, the chart number of each patient was encrypted and exclusively utilized for data linkage between different databases within the CGRD(12). Our study received approval from the Institutional Review Board of the Chang Gung Medical Foundation (IRB No.: 201702274B0), ensuring adherence to ethical guidelines and patient confidentiality. The CGRD's extensive collection of medical information made it an optimal resource for conducting retrospective clinical studies, greatly facilitating our research efforts.

©2024 Pan HC et al. *JAMA Network Open*.

Sensitivity, specificity, positive outcome controls, and negative outcome controls

To ensure the reliability of our findings, several sensitivity analyses were carried out. Firstly, we investigated the relationship between variables across different enrollment periods to account for possible changes in antihyperglycemic prescribing preferences over time. Additionally, we excluded patients with short follow-up durations and those who experienced mortality at different times during the follow-up period. Our study utilized global healthcare data from TriNetX, spanning from September 2002 to September 2022. Notably, between 2012 and 2015, the European Drug Administration (EMA) and the US Food and Drug Administration (FDA) approved three SGLT2 inhibitors (Kangliflozin, Daggliflozin, and Enggliflozin) for lowering blood glucose levels in type 2 diabetes patients. Considering the potential selection bias for patients in the non-SGLT2 inhibitors group before 2012, we conducted a distinct sensitivity analysis to address this concern. Moreover, we incorporated subjects who experienced events within 90 days post-discharge during the AKD phases in another sensitivity analysis. Secondly, diverse covariates were included in the Cox regression models to further validate the robustness of our results within each cohort. Furthermore, we performed specificity analyses to examine the beneficial effect of SGLT-2 inhibitors on different composite adverse outcomes. Thirdly, we utilized a new-user design focusing on those newly initiating SGLT-2i and juxtaposed this with individuals newly starting other active treatments, namely Sulfonylureas, dipeptidyl peptidase-4 inhibitors, or Pioglitazone.

To evaluate the effectiveness of our approach in reproducing known associations, we tested diabetes ketoacidosis and osteoporotic fractures as positive outcome controls. Additionally, we explored the correlation between SGLT-2 inhibitors treatment and five unrelated events (atopic dermatitis, conjunctivitis, melanoma, lymphoma, and Hodgkin's disease) as specified negative outcome controls. No prior evidence suggested a causal relationship between SGLT-2 inhibitors and the specified negative outcome controls. Sensitivity, specificity analysis, positive outcome controls and negative outcome controls were performed using R software (version 3.2.2, Free Software Foundation, Inc, Boston, MA), SAS software (version 9.2, SAS Inc., Cary, NC), and Stata/MP software (version 16, StataCorp, College Station, TX) s part of our rigorous analytical approach.

©2024 Pan HC et al. *JAMA Network Open*.

(A) Cohorts of type 2 diabetes with AKD who received and not received SGLT-2 inhibitors

1. Cohort of type 2 diabetes with AKD who received SGLT-2 inhibitors

This query was run on the network Research with 76 HCO(s) queried and 76 HCO(s) responded.

**Ungrouped terms**					
	must have		demographics	Age	Age (between 18 and 90 years (most recent occurrence))
**Group 1**					
	**Group 1A Discharge**				
	must have	any of	procedure	UMLS:CPT:99217	Observation care discharge day management (This code is to be utilized to report all services provided to a patient on discharge from outpatient hospital "observation status" if the discharge is on other than the initial date of "observation status." To report services to a patient designated as "observation status" or "inpatient status" and discharged on the same date, use the codes for Observation or Inpatient Care Services [including Admission and Discharge Services, 99234-99236 as appropriate.])
			procedure	UMLS:CPT:1013682	Hospital Discharge Services
			procedure	UMLS:CPT:99238	Hospital discharge day

©2024 Pan HC et al. *JAMA Network Open*.

				management; 30 minutes or less
		procedure	UMLS:CPT:99239	Hospital discharge day management; more than 30 minutes
date constraint		The terms in this group occurred on or before Sep 30, 2022		
event relationship		Any instance of dialysis+DM occurred within 3 months before or up to 1 day after any instance of Discharge		
**Group 1B dialysis+DM**				
must have	any of	procedure	UMLS:CPT:90937	Hemodialysis procedure requiring repeated evaluation(s) with or without substantial revision of dialysis prescription
		procedure	UMLS:CPT:90947	Dialysis procedure other than hemodialysis (eg, peritoneal dialysis, hemofiltration, or other continuous renal replacement therapies) requiring repeated evaluations by a physician or other qualified health care professional, with or without substantial revision of dialysis prescription
		procedure	UMLS:CPT:1012752	Hemodialysis Procedures
		diagnosis	UMLS:ICD10CM:E87	Other disorders of fluid, electrolyte and acid-base balance
		procedure	UMLS:CPT:90935	Hemodialysis procedure with single evaluation by a physician or other qualified health care professional
		procedure	UMLS:CPT:90945	Dialysis procedure other than

				hemodialysis (eg, peritoneal dialysis, hemofiltration, or other continuous renal replacement therapies), with single evaluation by a physician or other qualified health care professional
		procedure	UMLS:CPT:1006747	Hemodialysis Access, Intervascular Cannulation for Extracorporeal Circulation, or Shunt Insertion Procedures on Arteries and Veins
		procedure	UMLS:SNOMED:302497006	Hemodialysis
		procedure	UMLS:ICD9CM:39.95	Hemodialysis
		diagnosis	UMLS:ICD10CM:Z99.2	Dependence on renal dialysis
		procedure	UMLS:HCPCS:C1752	Catheter, hemodialysis/peritoneal, short-term
		procedure	UMLS:CPT:1012740	Dialysis Services and Procedures
	and	diagnosis	UMLS:ICD10CM:E11	Type 2 diabetes mellitus
**Group 2**				
**Group 2A Discharge**				
must have	any of	procedure	UMLS:CPT:99217	Observation care discharge day management (This code is to be utilized to report all services provided to a patient on discharge from outpatient hospital "observation status" if the discharge is on other than the initial date of "observation

				status." To report services to a patient designated as "observation status" or "inpatient status" and discharged on the same date, use the codes for Observation or Inpatient Care Services [including Admission and Discharge Services, 99234-99236 as appropriate.])
		procedure	UMLS:CPT:1013682	Hospital Discharge Services
		procedure	UMLS:CPT:99238	Hospital discharge day management; 30 minutes or less
		procedure	UMLS:CPT:99239	Hospital discharge day management; more than 30 minutes
date constraint		The terms in this group occurred at any time		
event relationship		Any instance of AKD occurred within 1 day and 3 months after any instance of Discharge		
**Group 2B AKD**				
must have		medication	NLM:ATC:A10BK	Sodium-glucose co-transporter 2 (SGLT2) inhibitors
cannot have		diagnosis	UMLS:ICD10CM:R69	Illness, unspecified
	or	diagnosis	UMLS:ICD10CM:R99-R99	Ill-defined and unknown cause of mortality (R99)
	or	demographics	Deceased	Deceased
	or	diagnosis	UMLS:ICD10CM:R99	Ill-defined and unknown cause of mortality
	or	procedure	UMLS:CPT:90937	Hemodialysis procedure

©2024 Pan HC et al. *JAMA Network Open*.

			requiring repeated evaluation(s) with or without substantial revision of dialysis prescription
or	procedure	UMLS:CPT:90947	Dialysis procedure other than hemodialysis (eg, peritoneal dialysis, hemofiltration, or other continuous renal replacement therapies) requiring repeated evaluations by a physician or other qualified health care professional, with or without substantial revision of dialysis prescription
or	procedure	UMLS:CPT:90945	Dialysis procedure other than hemodialysis (eg, peritoneal dialysis, hemofiltration, or other continuous renal replacement therapies), with single evaluation by a physician or other qualified health care professional
or	procedure	UMLS:CPT:1006747	Hemodialysis Access, Intervascular Cannulation for Extracorporeal Circulation, or Shunt Insertion Procedures on Arteries and Veins
or	procedure	UMLS:CPT:1012752	Hemodialysis Procedures
or	diagnosis	UMLS:ICD10CM:E87	Other disorders of fluid, electrolyte and acid-base balance
or	procedure	UMLS:ICD9CM:39.95	Hemodialysis

©2024 Pan HC et al. *JAMA Network Open*.

	or	procedure	UMLS:CPT:90935	Hemodialysis procedure with single evaluation by a physician or other qualified health care professional
	or	procedure	UMLS:SNOMED:302497006	Hemodialysis
	or	procedure	UMLS:CPT:1012740	Dialysis Services and Procedures
	or	procedure	UMLS:HCPCS:C1750	Catheter, hemodialysis/peritoneal, long-term
	or	diagnosis	UMLS:ICD10CM:Z99.2	Dependence on renal dialysis
	or	procedure	UMLS:HCPCS:C1752	Catheter, hemodialysis/peritoneal, short-term

2. **Cohort of type 2 diabetes with AKD who did not receive SGLT-2 inhibitors**

**Ungrouped terms**				
must have		demographics	Age	Age (between 18 and 90 years (most recent occurrence))
**Group 1**				
**Group 1A Discharge**				
must have	any of	procedure	UMLS:CPT:99217	Observation care discharge day management (This code is to be utilized to report all services provided to a patient on discharge from outpatient hospital "observation status" if the discharge is on other than

©2024 Pan HC et al. *JAMA Network Open*.

				the initial date of "observation status." To report services to a patient designated as "observation status" or "inpatient status" and discharged on the same date, use the codes for Observation or Inpatient Care Services [including Admission and Discharge Services, 99234-99236 as appropriate.])
		procedure	UMLS:CPT:1013682	Hospital Discharge Services
		procedure	UMLS:CPT:99238	Hospital discharge day management; 30 minutes or less
		procedure	UMLS:CPT:99239	Hospital discharge day management; more than 30 minutes
date constraint		The terms in this group occurred on or before Sep 30, 2022		
event relationship		Any instance of dialysis occurred within 3 months before or up to 1 day after any instance of Discharge		
**Group 1B dialysis**				
must have	any of	procedure	UMLS:CPT:90937	Hemodialysis procedure requiring repeated evaluation(s) with or without substantial revision of dialysis prescription
		procedure	UMLS:CPT:90947	Dialysis procedure other than hemodialysis (eg, peritoneal dialysis, hemofiltration, or other continuous renal replacement therapies) requiring repeated evaluations by a physician or other

		qualified health care professional, with or without substantial revision of dialysis prescription
procedure	UMLS:CPT:1012752	Hemodialysis Procedures
diagnosis	UMLS:ICD10CM:E87	Other disorders of fluid, electrolyte and acid-base balance
procedure	UMLS:CPT:90935	Hemodialysis procedure with single evaluation by a physician or other qualified health care professional
procedure	UMLS:CPT:90945	Dialysis procedure other than hemodialysis (eg, peritoneal dialysis, hemofiltration, or other continuous renal replacement therapies), with single evaluation by a physician or other qualified health care professional
procedure	UMLS:CPT:1006747	Hemodialysis Access, Intervascular Cannulation for Extracorporeal Circulation, or Shunt Insertion Procedures on Arteries and Veins
procedure	UMLS:SNOMED:302497006	Hemodialysis
procedure	UMLS:ICD9CM:39.95	Hemodialysis
diagnosis	UMLS:ICD10CM:Z99.2	Dependence on renal dialysis
procedure	UMLS:HCPCS:C1752	Catheter, hemodialysis/peritoneal, short-term

©2024 Pan HC et al. *JAMA Network Open*.

		procedure	UMLS:CPT:1012740	Dialysis Services and Procedures
	and	diagnosis	UMLS:ICD10CM:E11	Type 2 diabetes mellitus
**Group 2**				
**Group 2A Discharge**				
must have	any of	procedure	UMLS:CPT:99217	Observation care discharge day management (This code is to be utilized to report all services provided to a patient on discharge from outpatient hospital "observation status" if the discharge is on other than the initial date of "observation status." To report services to a patient designated as "observation status" or "inpatient status" and discharged on the same date, use the codes for Observation or Inpatient Care Services [including Admission and Discharge Services, 99234-99236 as appropriate.])
		procedure	UMLS:CPT:1013682	Hospital Discharge Services
		procedure	UMLS:CPT:99238	Hospital discharge day management; 30 minutes or less
		procedure	UMLS:CPT:99239	Hospital discharge day management; more than 30 minutes
date constraint		The terms in this group occurred at any time		
event relationship		Any instance of AKD occurred within 1 day and 3 months after any instance of Discharge		

©2024 Pan HC et al. *JAMA Network Open*.

Group 2B AKD				
cannot have		medication	NLM:ATC:A10BK	Sodium-glucose co-transporter 2 (SGLT2) inhibitors
	or	diagnosis	UMLS:ICD10CM:R69	Illness, unspecified
	or	diagnosis	UMLS:ICD10CM:R99-R99	Ill-defined and unknown cause of mortality (R99)
	or	demographics	Deceased	Deceased
	or	diagnosis	UMLS:ICD10CM:R99	Ill-defined and unknown cause of mortality
	or	procedure	UMLS:CPT:90937	Hemodialysis procedure requiring repeated evaluation(s) with or without substantial revision of dialysis prescription
	or	procedure	UMLS:CPT:90947	Dialysis procedure other than hemodialysis (eg, peritoneal dialysis, hemofiltration, or other continuous renal replacement therapies) requiring repeated evaluations by a physician or other qualified health care professional, with or without substantial revision of dialysis prescription
	or	procedure	UMLS:CPT:90945	Dialysis procedure other than hemodialysis (eg, peritoneal dialysis, hemofiltration, or other continuous renal replacement therapies), with single evaluation by a

©2024 Pan HC et al. *JAMA Network Open*.

			physician or other qualified health care professional
or	procedure	UMLS:CPT:1006747	Hemodialysis Access, Intervascular Cannulation for Extracorporeal Circulation, or Shunt Insertion Procedures on Arteries and Veins
or	procedure	UMLS:CPT:1012752	Hemodialysis Procedures
or	diagnosis	UMLS:ICD10CM:E87	Other disorders of fluid, electrolyte and acid-base balance
or	procedure	UMLS:ICD9CM:39.95	Hemodialysis
or	procedure	UMLS:CPT:90935	Hemodialysis procedure with single evaluation by a physician or other qualified health care professional
or	procedure	UMLS:SNOMED:302497006	Hemodialysis
or	procedure	UMLS:CPT:1012740	Dialysis Services and Procedures
or	procedure	UMLS:HCPCS:C1750	Catheter, hemodialysis/peritoneal, long-term
or	diagnosis	UMLS:ICD10CM:Z99.2	Dependence on renal dialysis
or	procedure	UMLS:HCPCS:C1752	Catheter, hemodialysis/peritoneal, short-term

(B) Outcome definition

**Mortality**		
**Outcome definition**		
Demographics	Deceased	Deceased
Diagnosis	UMLS:ICD10CM:R99	Ill-defined and unknown cause of mortality
Diagnosis	UMLS:ICD10CM:R99-R99	Ill-defined and unknown cause of mortality (R99)
Diagnosis	UMLS:ICD10CM:R69	Illness, unspecified

**MACE**		
**Outcome definition**		
Diagnosis	UMLS:ICD10CM:I63	Cerebral infarction
Diagnosis	UMLS:ICD10CM:I61	Nontraumatic intracerebral hemorrhage
Demographics	Deceased	Deceased
Diagnosis	UMLS:ICD10CM:R99	Ill-defined and unknown cause of mortality
Diagnosis	UMLS:ICD10CM:R69	Illness, unspecified
Diagnosis	UMLS:ICD10CM:I46	Cardiac arrest
Diagnosis	UMLS:ICD10CM:I21	Acute myocardial infarction
Diagnosis	UMLS:ICD10CM:I50	Heart failure

**MAKE**		
**Outcome definition**		
Procedure	UMLS:CPT:1012740	Dialysis Services and Procedures
Procedure	UMLS:CPT:90945	Dialysis procedure other than hemodialysis (eg, peritoneal dialysis, hemofiltration, or other continuous renal replacement therapies), with

©2024 Pan HC et al. *JAMA Network Open*.

		single evaluation by a physician or other qualified health care professional
Diagnosis	UMLS:ICD10CM:Z99.2	Dependence on renal dialysis
Demographics	Deceased	Deceased
Diagnosis	UMLS:ICD10CM:R99	Ill-defined and unknown cause of mortality
Diagnosis	UMLS:ICD10CM:R99-R99	Ill-defined and unknown cause of mortality (R99)
Diagnosis	UMLS:ICD10CM:R69	Illness, unspecified
Procedure	UMLS:ICD9CM:39.95	Hemodialysis
Procedure	UMLS:CPT:1012752	Hemodialysis Procedures
Procedure	UMLS:CPT:1006747	Hemodialysis Access, Intervascular Cannulation for Extracorporeal Circulation, or Shunt Insertion Procedures on Arteries and Veins

ketoacidosis

Outcome definition

Diagnosis	UMLS:ICD10CM:E08.10	Diabetes mellitus due to underlying condition with ketoacidosis without coma
Diagnosis	UMLS:ICD10CM:E11.11	Type 2 diabetes mellitus with ketoacidosis with coma
Diagnosis	UMLS:ICD10CM:E10.10	Type 1 diabetes mellitus with ketoacidosis without coma
Diagnosis	UMLS:ICD10CM:E08.10	Diabetes mellitus due to underlying condition with ketoacidosis without coma
Diagnosis	UMLS:ICD10CM:E13.1	Other specified diabetes mellitus with ketoacidosis
Diagnosis	UMLS:ICD10CM:E10.1	Type 1 diabetes mellitus with ketoacidosis
Diagnosis	UMLS:ICD10CM:E11.1	Type 2 diabetes mellitus with ketoacidosis
Diagnosis	UMLS:ICD10CM:E11.10	Type 2 diabetes mellitus with ketoacidosis without coma
Diagnosis	UMLS:ICD10CM:E08.1	Diabetes mellitus due to underlying condition with ketoacidosis
Diagnosis	UMLS:ICD10CM:E11.11	Type 2 diabetes mellitus with ketoacidosis with

©2024 Pan HC et al. *JAMA Network Open*.

		coma
Diagnosis	UMLS:ICD10CM:E10.11	Type 1 diabetes mellitus with ketoacidosis with coma
Diagnosis	UMLS:ICD10CM:E09.10	Drug or chemical induced diabetes mellitus with ketoacidosis without coma
Diagnosis	UMLS:ICD10CM:E09.1	Drug or chemical induced diabetes mellitus with ketoacidosis
Diagnosis	UMLS:ICD10CM:E13.11	Other specified diabetes mellitus with ketoacidosis with coma
Diagnosis	UMLS:ICD10CM:E09.11	Drug or chemical induced diabetes mellitus with ketoacidosis with coma
Diagnosis	UMLS:ICD10CM:E08.11	Diabetes mellitus due to underlying condition with ketoacidosis with coma

Osteoporotic fracture

Outcome definition

Diagnosis	UMLS:ICD10CM:M81.0	Age-related osteoporosis without current pathological fracture

Atopic dermatitis

Outcome definition

Diagnosis	UMLS:ICD10CM:L20	Atopic dermatitis
Diagnosis	UMLS:ICD10CM:L20.89	Other atopic dermatitis
Diagnosis	UMLS:ICD10CM:L20.81	Atopic neurodermatitis
Diagnosis	UMLS:ICD10CM:L25	Unspecified contact dermatitis
Diagnosis	UMLS:ICD10CM:L85.3	Xerosis cutis

Conjunctivitis

©2024 Pan HC et al. *JAMA Network Open*.

Outcome definition		
Diagnosis	UMLS:ICD10CM:H16	Keratitis

Melanoma

Outcome definition		
Diagnosis	UMLS:ICD10CM:D03	Melanoma in situ

Lymphoma

Outcome definition		
Diagnosis	UMLS:ICD10CM:C81-C96	Malignant neoplasms of lymphoid, hematopoietic and related tissue

Hodgkin

Outcome definition		
Diagnosis	UMLS:ICD10CM:C81	Hodgkin lymphoma
Diagnosis	UMLS:ICD10CM:C81.9	Hodgkin lymphoma, unspecified
Diagnosis	UMLS:ICD10CM:C81.90	Hodgkin lymphoma, unspecified, unspecified site
Diagnosis	UMLS:ICD10CM:C81.99	Hodgkin lymphoma, unspecified, extranodal and solid organ sites

Mortality + HF

Outcome definition		
Demographics	Deceased	Deceased
Diagnosis	UMLS:ICD10CM:R99	Ill-defined and unknown cause of mortality
Diagnosis	UMLS:ICD10CM:R69	Illness, unspecified
Diagnosis	UMLS:ICD10CM:I50	Heart failure
Diagnosis	UMLS:ICD10CM:I50.9	Heart failure, unspecified
Diagnosis	UMLS:ICD10CM:I50.1	Left ventricular failure, unspecified
Diagnosis	UMLS:ICD10CM:I50.3	Diastolic (congestive) heart failure

©2024 Pan HC et al. *JAMA Network Open*.

Diagnosis	UMLS:ICD10CM:I50.2	Systolic (congestive) heart failure
Diagnosis	UMLS:ICD10CM:I50.32	Chronic diastolic (congestive) heart failure
Diagnosis	UMLS:ICD10CM:I50.22	Chronic systolic (congestive) heart failure
Diagnosis	UMLS:ICD10CM:I50.21	Acute systolic (congestive) heart failure
Diagnosis	UMLS:ICD10CM:I50.31	Acute diastolic (congestive) heart failure
Diagnosis	UMLS:ICD10CM:I50.30	Unspecified diastolic (congestive) heart failure
Diagnosis	UMLS:ICD10CM:I11.0	Hypertensive heart disease with heart failure

Mortality + MI

Outcome definition

Diagnosis	UMLS:ICD10CM:I63	Cerebral infarction
Procedure	UMLS:SNOMED:281572005	Direct current cardiac shock
Demographics	Deceased	Deceased
Diagnosis	UMLS:ICD10CM:R99	Ill-defined and unknown cause of mortality
Diagnosis	UMLS:ICD10CM:R99-R99	Ill-defined and unknown cause of mortality (R99)
Diagnosis	UMLS:ICD10CM:R69	Illness, unspecified
Diagnosis	UMLS:ICD10CM:I21	Acute myocardial infarction
Demographics	Deceased	Deceased
Diagnosis	UMLS:ICD10CM:R99	Ill-defined and unknown cause of mortality
Diagnosis	UMLS:ICD10CM:R99-R99	Ill-defined and unknown cause of mortality (R99)
Diagnosis	UMLS:ICD10CM:R69	Illness, unspecified

Mortality + Stroke

Outcome definition

Demographics	Deceased	Deceased
Diagnosis	UMLS:ICD10CM:R99	Ill-defined and unknown cause of mortality
Diagnosis	UMLS:ICD10CM:R69	Illness, unspecified
Diagnosis	UMLS:ICD10CM:I63	Cerebral infarction
Diagnosis	UMLS:ICD10CM:I61	Nontraumatic intracerebral hemorrhage

©2024 Pan HC et al. *JAMA Network Open*.

eTable 1. Presumptive Causes of AKI

	All patients (n=230366)	SGLT-2i group (n=5319)	Control group (n=225047)	*P-value*
Cardiogenic shock	71207 (30.9%)	2792 (52.5%)	68414 (30.4%)	< 0.01
Cardiorenal syndrome	89636 (38.9%)	3218 (60.5%)	86418 (38.4%)	< 0.01
Sepsis without septic shock	146282 (63.5%)	3378 (63.5%)	142905 (63.5%)	0.11
Septic shock	42109 (18.3%)	926 (17.4%)	41184 (18.3%)	0.01
Hypovolemic shock	5073 (2.2%)	122 (2.3%)	4951 (2.2%)	0.11
Obstructive uropathy	26437 (11.5%)	782 (14.7%)	25655 (11.4%)	0.10
Drug-related AKI or Contrast nephropathy	16356 (7.1%)	378 (7.1%)	15978 (7.0%)	0.95
Others*	17281 (7.5%)	628 (11.8%)	16653 (7.4%)	< 0.01

Abbreviation: AKI, acute kidney injury; SGLT-2i, sodium–glucose cotransporter 2 inhibitors

*Others: hypertension crisis, postpartum AKI, etc

©2024 Pan HC et al. *JAMA Network Open*.

eTable 2. Kidney Function and Electrolytes After Withdrawal of Dialysis

	SGLT-2i group (n=5319)	Control group (n=225047)	*P-value*
eGFR, mL/min/1.73m2	69.8 ± 30.9	68.1 ± 39.4	< 0.01
Sodium, mEq/L	138.0 ± 3.4	138.0 ± 4.2	< 0.01
Potassium, mEq/L	4.2 ± 0.5	4.2 ± 0.6	< 0.01

Abbreviation: eGFR, estimated glomerular filtration rate; SGLT-2i, sodium–glucose cotransporter 2 inhibitors

©2024 Pan HC et al. *JAMA Network Open*.

eTable 3. Risk of Mortality in Patients With Type 2 Diabetes and AKD: Comparison Between SGLT-2I Users and Nonusers After Propensity Score Matching

The table presents results from a cohort analysis after propensity score matching. Cohort 1 consist of type 2 diabetes patients with AKD who received SGLT-2 inhibitors, while cohort 2 comprises those who did not receive of SGLT-2 inhibitors.

Mortality

Risk analysis

Cohort	Patients in cohort	Patients with outcome	Risk
1 (new)DM+AKD+SGLT2	5,317	481	0.090
2 (new)DM+AKD-SGLT2	5,317	994	0.187

		95% CI	z	p
**Risk Difference**	-0.096	(-0.109, -0.083)	-14.393	0.000
**Risk Ratio**	0.484	(0.437, 0.536)	N/A	N/A
**Odds Ratio**	0.433	(0.385, 0.486)	N/A	N/A

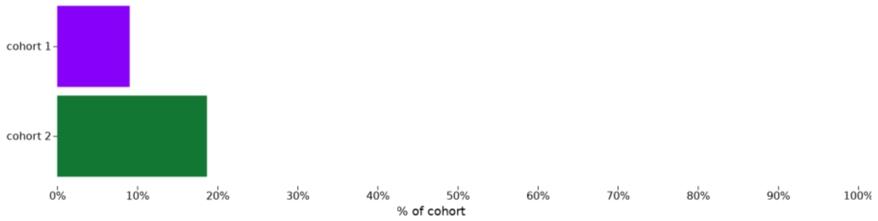

©2024 Pan HC et al. *JAMA Network Open*.

Kaplan - Meier survival analysis

Cohort	Patients in cohort	Patients with outcome	Median survival (days)	Survival probability at end of time window
1 (new)DM+AKD+SGLT2	5,317	481	--	72.63%
2 (new)DM+AKD-SGLT2	5,317	994	--	65.56%

	χ^2	df	p
**Log-Rank Test**	44.707	1	0.000

	Hazard Ratio	95% CI	χ^2	df	p
**Hazard Ratio and Proportionality**	0.687	(0.615, 0.767)	3.820	1	0.051

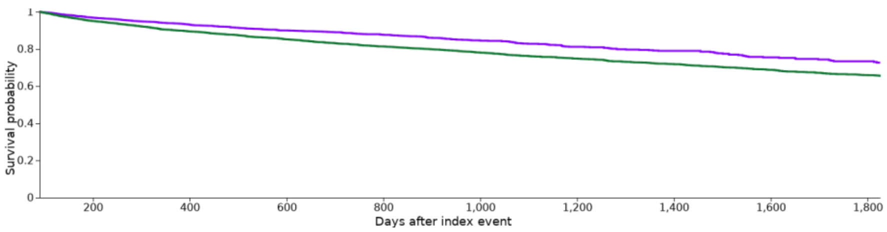

Number of instances

Cohort	Patients in cohort	Patients with outcome	Mean	Standard Deviation	Median
1 (new)DM+AKD+SGLT2	5,317	481	1.917	2.321	1
2 (new)DM+AKD-SGLT2	5,317	994	1.697	1.725	1

©2024 Pan HC et al. *JAMA Network Open*.

	t	df	p
**Test Statistics**	2.039	1473	0.042

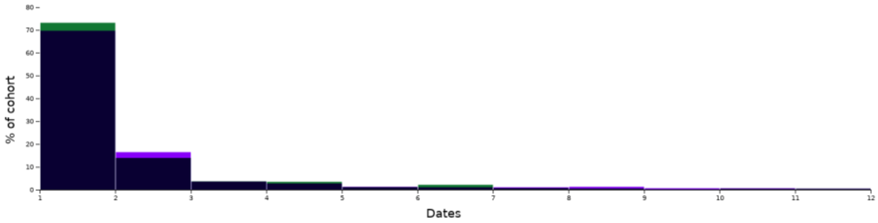

©2024 Pan HC et al. *JAMA Network Open*.

eTable 4. Risk of MAKE in Patients With Type 2 Diabetes and AKD: Comparison Between SGLT-2I Users and Nonusers After Propensity Score Matching

The table presents results from a cohort analysis after propensity score matching. Patients with a history of MAKE were part of the baseline cohort but were excluded from this analysis. Cohort 1 consist of type 2 diabetes patients with AKD who received SGLT-2 inhibitors, while cohort 2 comprises those who did not receive of SGLT-2 inhibitors.

MAKE

Risk analysis

Cohort	Patients in cohort	Patients with outcome	Risk
1 (new)DM+AKD+SGLT2	5,317	504	0.095
2 (new)DM+AKD-SGLT2	5,317	1,119	0.210

		95% CI	z	p
**Risk Difference**	-0.116	(-0.129, -0.102)	-16.584	0.000
**Risk Ratio**	0.450	(0.408, 0.497)	N/A	N/A
**Odds Ratio**	0.393	(0.351, 0.440)	N/A	N/A

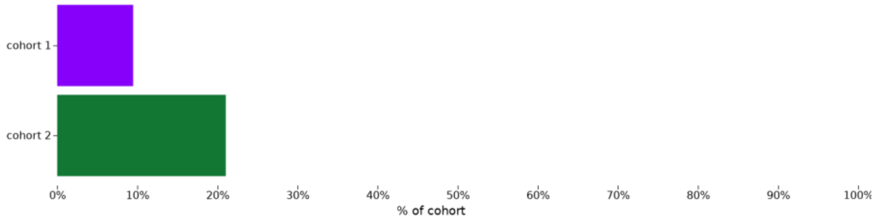

©2024 Pan HC et al. *JAMA Network Open*.

Kaplan – Meier survival analysis

Cohort	Patients in cohort	Patients with outcome	Median survival (days)	Survival probability at end of time window
1 (new)DM+AKD+SGLT2	5,317	504	--	71.68%
2 (new)DM+AKD-SGLT2	5,317	1,119	--	62.02%

	χ^2	df	p
**Log-Rank Test**	77.348	1	0.000

	Hazard Ratio	95% CI	χ^2	df	p
**Hazard Ratio and Proportionality**	0.622	(0.559, 0.692)	5.880	1	0.015

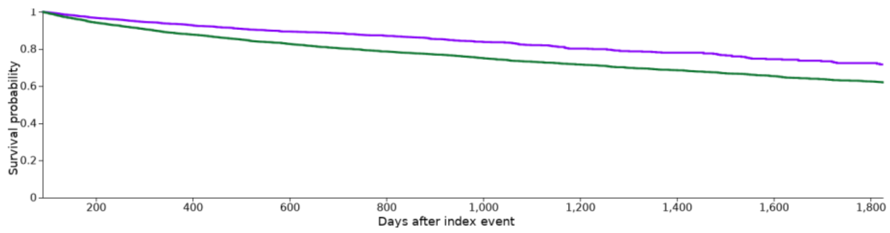

Number of instances

Cohort	Patients in cohort	Patients with outcome	Mean	Standard Deviation	Median
1 (new)DM+AKD+SGLT2	5,317	504	2.306	3.821	1
2 (new)DM+AKD-SGLT2	5,317	1,119	2.441	6.687	1

©2024 Pan HC et al. *JAMA Network Open*.

	t	df	p
**Test Statistics**	-0.426	1621	0.670

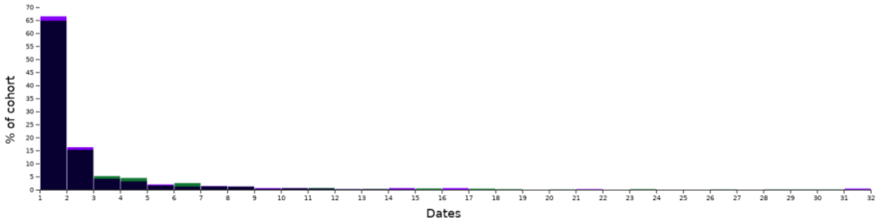

©2024 Pan HC et al. *JAMA Network Open*.

eTable 5. Comparing SGLT-2I Users and Nonusers in Relation to MACE

	Before matching			After matching		
	**SGLT-2i group (n=5535)**	**Control group (n=232821)**	**Std diff**	**SGLT-2i group (n=1732)**	**Control group (n=2670)**	**Std diff**
**Demographic**						
Age, mean ± SD	64.0 ± 12.3	67.5 ± 15.3	0.26	64.0 ± 12.3	64.2 ± 13.9	0.02
Male, n (%)	2848 (59.9%)	104293 (52.9%)	0.14	2827 (59.8%)	2852 (60.3%)	0.01
White, n (%)	2998 (63.1%)	127397 (64.6%)	0.03	2980 (63.1%)	3010 (63.7%)	0.01
**Comorbidities, n (%)**						
Hyperlipidemia	2516 (52.9%)	34942 (17.7%)	0.79	2497 (52.8%)	2129 (45.0%)	0.16
Chronic kidney disease	1234 (26.0%)	20765 (10.5%)	0.41	1222 (25.9%)	1154 (24.4%)	0.03
Hyperuricemia	160 (3.4%)	2876 (1.5%)	0.13	158 (3.3%)	184 (3.9%)	0.03
Cerebrovascular diseases	697 (14.7%)	11240 (5.7%)	0.30	689 (14.6%)	628 (13.3%)	0.04
Overweight	1804 (37.9%)	20907 (10.6%)	0.67	1782 (37.7%)	1744 (36.9%)	0.02
COPD	651 (13.7%)	9992 (5.1%)	0.30	641 (13.6%)	637 (13.5%)	0.002
Musculoskeletal disease	2718 (57.2%)	54027 (27.4%)	0.63	2694 (57.0%)	2730 (57.8%)	0.02
Malignancy	64 (1.3%)	1676 (0.8%)	0.05	64 (1.4%)	72 (1.5%)	0.01
**Medication, n (%)**						
Metformin	2054 (43.2%)	25814 (13.1%)	0.71	2031 (43.0%)	2048 (43.3%)	0.007
Sulfonylureas	892 (18.8%)	13574 (6.9%)	0.36	883 (18.7%)	895 (18.9%)	0.006
DPP4i	645 (11.7%)	6176 (2.7%)	0.35	641 (11.7%)	439 (8.0%)	0.12
Acarbose	13 (0.3%)	138 (0.1%)	0.049	13 (0.3%)	10 (0.2%)	0.01
GLP-1 analogues	747 (15.7%)	3443 (1.7%)	0.51	722 (15.3%)	665 (14.1%)	0.03
Insulin	3054 (64.2%)	34701 (17.6%)	1.08	3026 (64.0%)	3090 (65.4%)	0.03
Aspirin	2102 (44.2%)	24436 (12.4%)	0.76	2077 (43.9%)	2089 (44.2%)	0.005
Clopidogrel	701 (14.7%)	7342 (3.7%)	0.39	691 (14.6%)	604 (12.8%)	0.05
Atorvastatin	2088 (43.9%)	20412 (10.3%)	0.82	2063 (43.7%)	2094 (44.3%)	0.01
Allopurinol	322 (6.8%)	4706 (2.4%)	0.21	317 (6.7%)	276 (5.8%)	0.04
Febuxostat	12 (0.3%)	262 (0.1%)	0.03	12 (0.3%)	18 (0.4%)	0.02
Alpha-blocker	612 (12.9%)	8946 (4.5%)	0.30	608 (12.9%)	588 (12.4%)	0.01
Beta-blocker	2728 (57.4%)	37872 (19.2%)	0.85	2702 (57.2%)	2379 (50.3%)	0.14
CCB	1624 (34.2%)	25417 (12.9%)	0.52	1609 (34.0%)	1515 (32.1%)	0.04

©2024 Pan HC et al. *JAMA Network Open*.

ACEI or ARB	2982 (62.7%)	40778 (20.7%)	0.94	2959 (62.6%)	2543 (53.8%)	0.18
**Laboratory**						
BMI	32.7 ± 7.1	31.3 ± 7.1	0.19	32.7 ± 7.1	32.4 ± 7.4	0.04
30-60 kg/m^2	1193 (25.1%)	21930 (11.1%)	0.37	1185 (25.1%)	1148 (24.3%)	0.02
25-30 kg/m^2	622 (13.1%)	13403 (6.8%)	0.21	615 (13.0%)	628 (13.3%)	0.008
5-25 kg/m^2	355 (6.4%)	9678 (4.2%)	0.10	354 (6.4%)	352 (6.4%)	0.001
WBC, x10^3/uL	8.2 ± 3.7	9.8 ± 76.6	0.03	8.2 ± 3.7	8.8 ± 4.5	0.15
Platelet, x10^3/uL	244.3 ± 94.8	241.8 ± 102.2	0.03	244.2 ± 94.9	255.2 ± 108.2	0.11
eGFR, mL/min/1.73m^2	74.0 ± 32.4	70.4 ± 35.8	0.11	74.0 ± 32.4	72.4 ± 37.8	0.05
Total cholesterol, mg/dL	160.3 ± 62.2	165.9 ± 51.4	0.10	160.4 ± 62.3	160.8 ± 54.3	0.007
HbA1C	8.4 ± 2.2	7.5 ± 2.0	0.41	8.4 ± 2.2	8.2 ± 2.1	0.08
7.5-12.0%	1768 (37.2%)	18155 (9.2%)	0.70	1744 (36.9%)	1761 (37.3%)	0.007
6.5-7.5%	1211 (21.9%)	17538 (7.7%)	0.41	1190 (21.6%)	1208 (22.0%)	0.008
5.0-6.5%	686 (14.4%)	19033 (9.6%)	0.15	685 (14.5%)	703 (14.9%)	0.01
AST, units/L	34.5 ± 268.7	32.0 ± 92.5	0.01	34.0 ± 268.3	32.0 ± 68.0	0.01
Sodium, mEq/L	137.7 ± 3.4	138.1 ± 3.8	0.12	137.7 ± 3.4	137.7 ± 3.8	0.01
Potassium, mEq/L	4.1 ± 0.5	4.2 ± 0.5	0.10	4.1 ± 0.5	4.2 ± 0.5	0.14
BNP, pg/mL	806.3 ± 2408.0	773.8 ± 2828.6	0.01	819.7 ± 2438.7	640.9 ± 1800.6	0.08

Abbreviations: ACEI, angiotensin converting enzyme inhibitors; ARB, angiotensin receptor blockers**;** AST, aspartate transaminase; BMI, body mass index; BNP, B-type natriuretic peptite; CCB, calcium channel blocker**;** COPD, chronic obstructive pulmonary disease; DPP4i, Dipeptidyl peptidase-4 inhibitor; eGFR, estimated, glomerular filtration rate; GLP-1; glucagon-like peptide 1; MACE, major adverse cardiac event; SD, standard deviation; SGLT-2i, sodium–glucose cotransporter 2 inhibitors

©2024 Pan HC et al. *JAMA Network Open*.

eTable 6. Risk of MACE in Patients With Type 2 Diabetes and AKD: Comparison Between SGLT-2I Users and Nonusers After Propensity Score Matching

The table presents results from a cohort analysis after propensity score matching. Patients with a history of MACE were excluded from the analysis. Cohort 1 consist of type 2 diabetes patients with AKD who received SGLT-2 inhibitors, while cohort 2 comprises those who did not receive of SGLT-2 inhibitors.

MACE

Risk analysis excluding patients with outcome prior to the time window

Cohort	Patients in cohort	Patients with outcome	Risk
1 (new)DM+AKD+SGLT2	1,732	233	0.135
2 (new)DM+AKD-SGLT2	2,670	690	0.258

		95% CI	z	p
**Risk Difference**	-0.124	(-0.147, -0.101)	-9.865	0.000
**Risk Ratio**	0.521	(0.455, 0.596)	N/A	N/A
**Odds Ratio**	0.446	(0.379, 0.525)	N/A	N/A

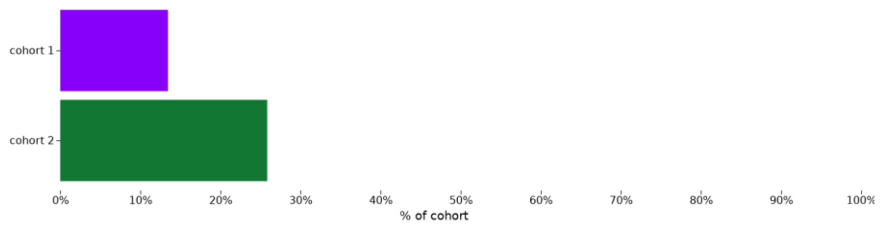

3,765 patients in Cohort 1 and 2,827 patients in Cohort 2 were excluded from results because they had the outcome prior to the time window.

©2024 Pan HC et al. *JAMA Network Open*.

Kaplan - Meier survival analysis excluding patients with outcome prior to the time window

Cohort	Patients in cohort	Patients with outcome	Median survival (days)	Survival probability at end of time window
1 (new)DM+AKD+SGLT2	1,732	233	--	62.58%
2 (new)DM+AKD-SGLT2	2,670	690	--	52.64%

	χ^2	df	p
**Log-Rank Test**	13.736	1	0.000

	Hazard Ratio	95% CI	χ^2	df	p
**Hazard Ratio and Proportionality**	0.754	(0.649, 0.876)	0.269	1	0.604

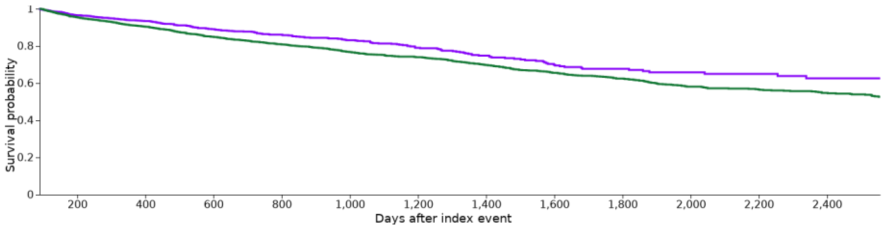

3,765 patients in Cohort 1 and 2,827 patients in Cohort 2 were excluded from results because they had the outcome prior to the time window.

Number of instances excluding patients with outcome prior to the time window

Cohort	Patients in cohort	Patients with outcome	Mean	Standard Deviation	Median

©2024 Pan HC et al. *JAMA Network Open*.

1	(new)DM+AKD+SGLT2	1,732	233	4.052	5.928	2
2	(new)DM+AKD-SGLT2	2,670	690	5.122	10.689	2

	t	df	p
**Test Statistics**	-1.454	921	0.146

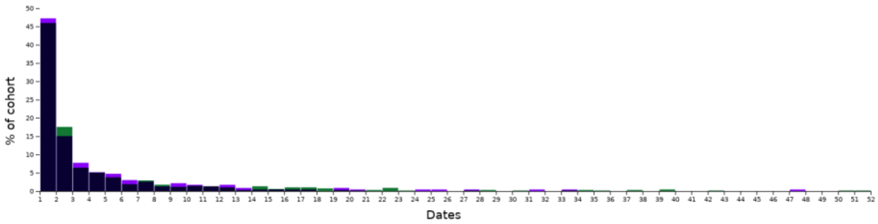

©2024 Pan HC et al. *JAMA Network Open*.

eTable 7. Sensitivity Analysis for All-Cause Mortality Between SLGT-2I Users and Nonusers

	aHR (95%CI)
**All eligible subjects without weighting (n=658,254)**	0.62 (0.55-0.69)
1:1 PSM, caliper=0.2	0.69 (0.62-0.77)
**Eligible subjects with different enrolled period, 1:1 PSM**	
Patient enrolled since 2012	0.65 (0.57-0.73)
Patient enrolled in before 2020	0.40 (0.33-0.49)
Patients enrolled between 2020 and 2022	0.50 (0.42-0.59)
Follow up 1 year	0.72 (0.58-0.88)
Follow up 2 year	0.53 (0.45-0.62)
Follow up 3 year	0.66 (0.57-0.76)
**Eligible subjects with different outcome definition, 1:1 PSM**	
Include died within 3 months after discharge	0.69 (0.61-0.77)
**Cox regression models with different covariates**	
Model 1 (age and gender, ethnicity)	0.68 (0.60-0.78)
Model 2 (age, gender, ethnicity, comorbidities)	0.65 (0.57-0.74)
Model 3 (age, gender, ethnicity, comorbidities, Na)	0.56 (0.50-0.63)
Model 4 (age, gender, ethnicity, comorbidities, Na, K)	0.58 (0.51-0.65)
Model 5 (age, gender, ethnicity, comorbidities, Na, K, hyperlipidemia)	0.60 (0.54-0.68)
Model 6 (age, gender, ethnicity, comorbidities, Na, K, hyperlipidemia, use of DPP4i)	0.61 (0.54-0.68)
Model 7 (age, gender, ethnicity, comorbidities, Na, K, hyperlipidemia, use of DPP4i and ACEI/ARB)	0.63 (0.56-0.71)
Model 8 (age, gender, ethnicity, comorbidities, Na, K, hyperlipidemia, use of DPP4i, ACEI/ARB, and GLP-1 agonists)	0.61 (0.54-0.68)
Model 9 (final full model)	0.59 (0.52-0.67)

Abbreviations: ACEI, angiotensin converting enzyme inhibitors; aHR, adjusted hazard ratio; ARB, CI, confidence interval; DPP4i, Dipeptidyl peptidase-4 inhibitor; GLP-1; glucagon-like peptide 1; PSM, propensity score matching; SGLT-2i, sodium–glucose cotransporter 2 inhibitor

©2024 Pan HC et al. *JAMA Network Open*.

eTable 8. Sensitivity Analysis for All-Cause Mortality, MAKE, and MACE Between SLGT-2I Users and Other Active Treatment (Sulfonylureas, Dipeptidyl Peptidase-4 Inhibitor, or Pioglitazone) Users in a New-User Design

**Outcome**	**Patients with outcome**		**aHR (95%CI)**
	**SGLT-2i group**	**Other active treatment group**	
**Primary outcome**			
Mortality	7.5% (313/4164)	16.5% (689/4164)	0.77 (0.68-0.89)
**Secondary outcome**			
MAKE	9.3% (387/4164)	21.9% (910/4164)	0.65 (0.58-0.74)
MACE	16.3% (161/989)	29.6% (528/1785)	0.82 (0.68-0.98)

Abbreviations: aHR, adjusted hazard ratios; CI, confidence interval; MACE, major adverse cardiac events; MAKE, major adverse kidney events; SGLT-2i, sodium–glucose cotransporter 2 inhibitor

©2024 Pan HC et al. *JAMA Network Open*.

eFigure 1. Positive Outcome Control, Negative Outcome Control, and Specificity Analysis

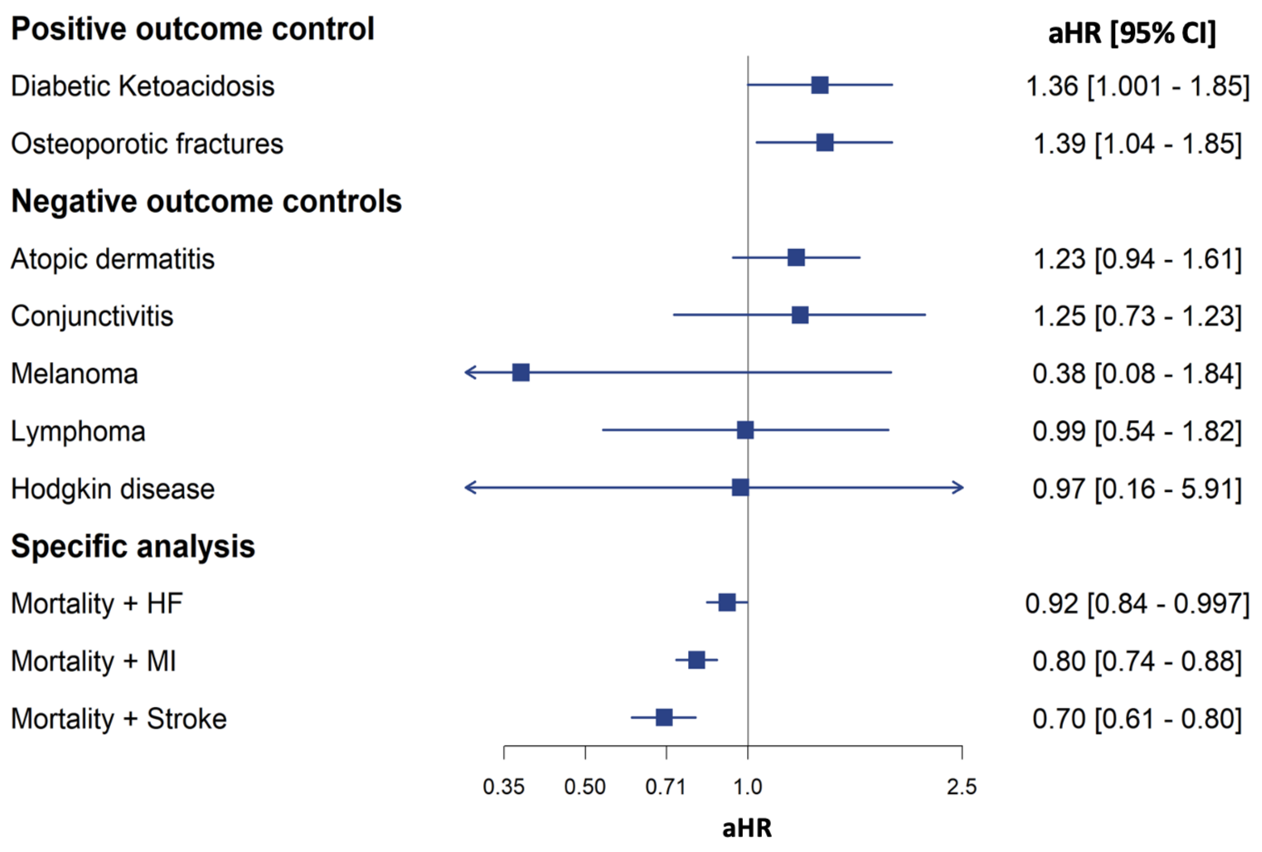

Abbreviations: CI, confidence interval; DPP4i, ESRD, end-stage renal disease; HF, heart failure; HR, hazard ratio; MI, myocardial infarction; SGLT-2i, sodium–glucose cotransporter 2 inhibitor

©2024 Pan HC et al. *JAMA Network Open*.

©2024 Pan HC et al. *JAMA Network Open*.

eFigure 2. External Validation by CGRD Database

The Kaplan-Meier curves presented the long-term outcomes of interest, including (A) all-cause mortality (B) MAKE (C) MACE

(A)

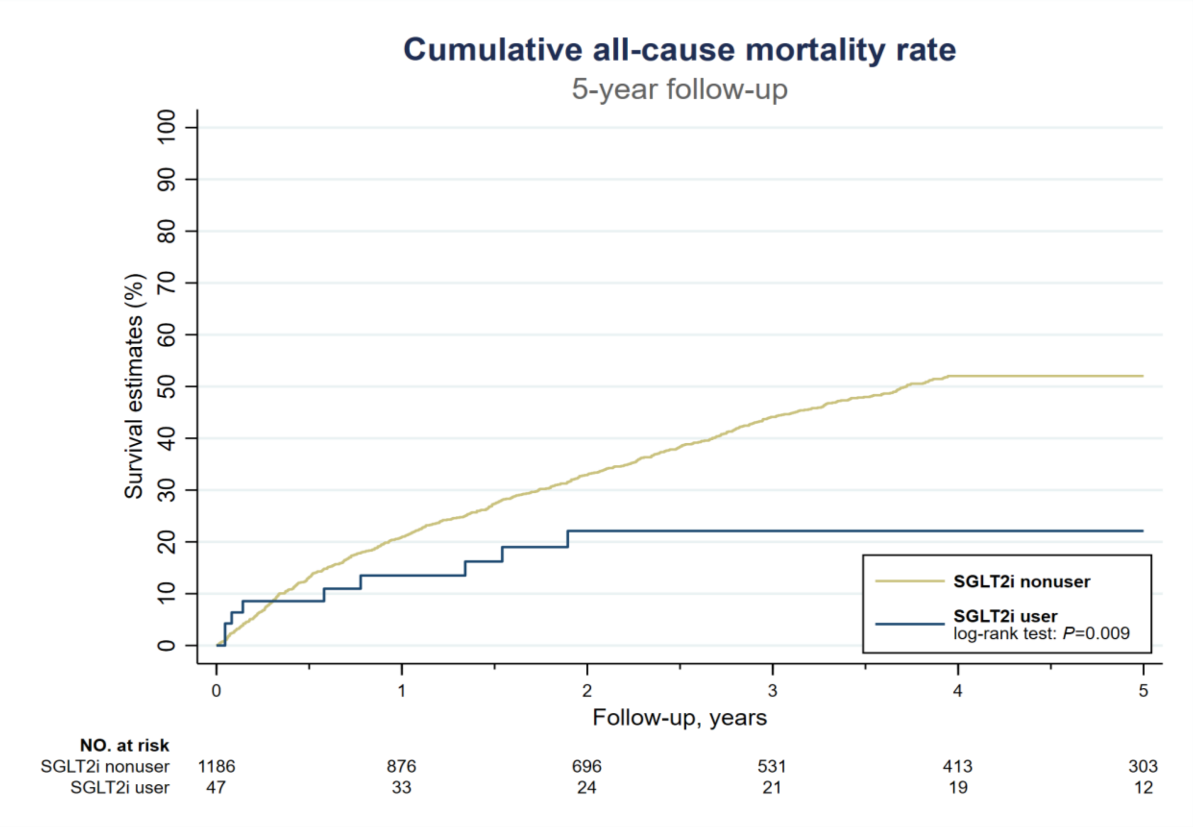

(B)

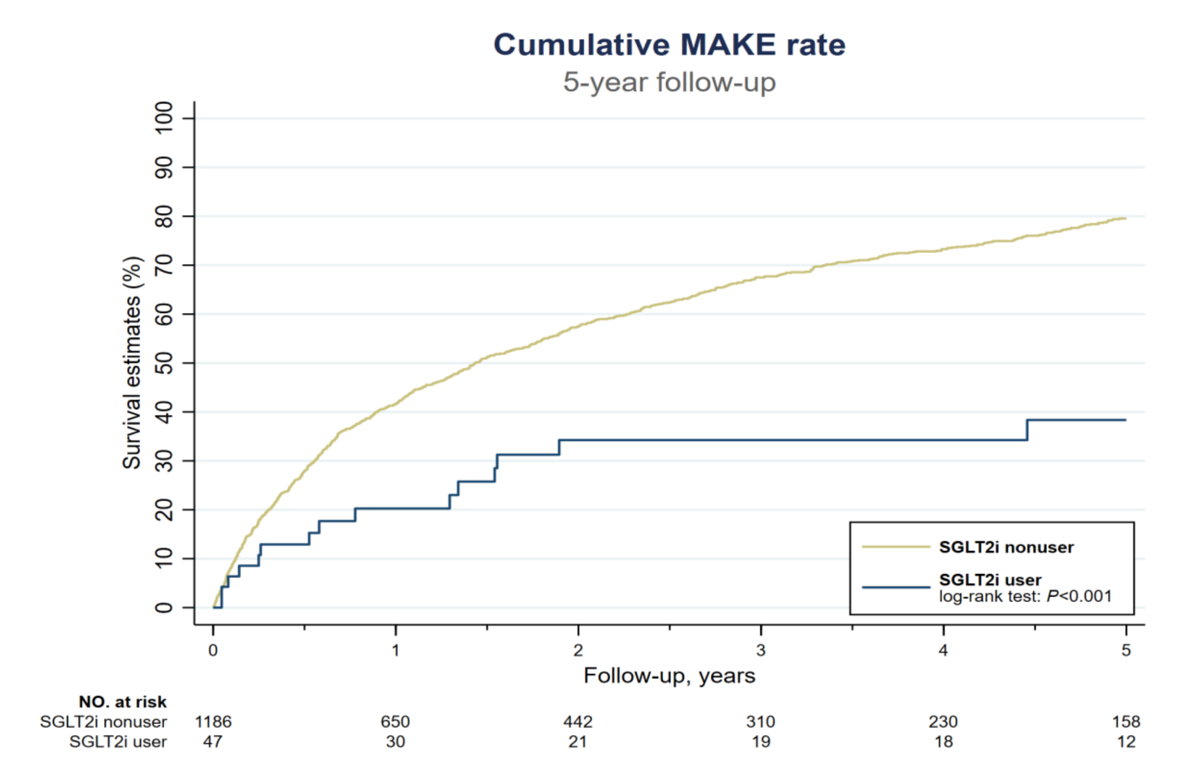

©2024 Pan HC et al. *JAMA Network Open*.

(C)

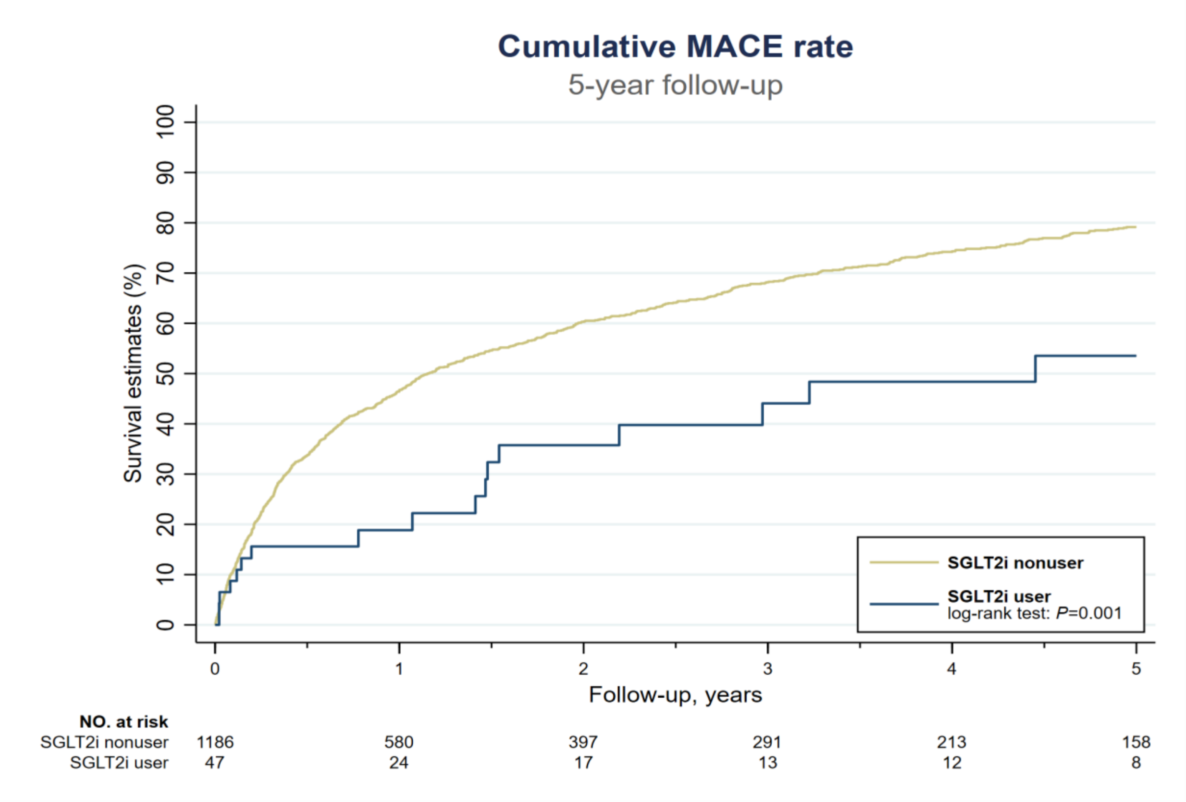

Abbreviations: AKD, acute kidney disease; CGRD, Chang Gung Research Database; MACE, major adverse cardiac event; MAKE, major adverse kidney event; SGLT-2i, sodium–glucose cotransporter 2 inhibitor

©2024 Pan HC et al. *JAMA Network Open*.

eReferences

1. Topaloglu U, Palchuk MB. Using a Federated Network of Real-World Data to Optimize Clinical Trials Operations. JCO Clin Cancer Inform. 2018;2:1-10.
2. MacKenzie SL, Wyatt MC, Schuff R, Tenenbaum JD, Anderson N. Practices and perspectives on building integrated data repositories: results from a 2010 CTSA survey. J Am Med Inform Assoc. 2012;19(e1):e119-24.
3. Hudson CL, Topaloglu U, Bian J, Hogan W, Kieber-Emmons T. Automated Tools for Clinical Research Data Quality Control using NCI Common Data Elements. AMIA Jt Summits Transl Sci Proc. 2014;2014:60-9.
4. Kahn MG, Brown JS, Chun AT, Davidson BN, Meeker D, Ryan PB, et al. Transparent reporting of data quality in distributed data networks. EGEMS (Wash DC). 2015;3(1):1052.
5. Weiskopf NG, Hripcsak G, Swaminathan S, Weng C. Defining and measuring completeness of electronic health records for secondary use. J Biomed Inform. 2013;46(5):830-6.
6. Shao SC, Chan YY, Kao Yang YH, Lin SJ, Hung MJ, Chien RN, et al. The Chang Gung Research Database—a multi-institutional electronic medical records database for real-world epidemiological studies in Taiwan. Pharmacoepidemiology and drug safety. 2019;28(5):593-600.
7. Shao S-C, Chang K-C, Hung M-J, Yang N-I, Chan Y-Y, Chen H-Y, et al. Comparative risk evaluation for cardiovascular events associated with dapagliflozin vs. empagliflozin in real-world type 2 diabetes patients: a multi-institutional cohort study. Cardiovascular diabetology. 2019;18(1):1-15.
8. Shao S-C, Chang K-C, Lin S-J, Chien R-N, Hung M-J, Chan Y-Y, et al. Favorable pleiotropic effects of sodium glucose cotransporter 2 inhibitors: head-to-head comparisons with dipeptidyl peptidase-4 inhibitors in type 2 diabetes patients. Cardiovascular diabetology. 2020;19(1):1-11.
9. Lin FJ, Wang CC, Hsu CN, Yang CY, Wang CY, Ou HT. Renoprotective effect of SGLT-2 inhibitors among type 2 diabetes patients with different baseline kidney function: a multi-center study. Cardiovasc Diabetol. 2021;20(1):203.
10. Shao S-C, Chang K-C, Lin S-J, Chang S-H, Hung M-J, Chan Y-Y, et al. Differences in outcomes of hospitalizations for heart failure after SGLT2 inhibitor treatment: effect modification by atherosclerotic cardiovascular disease. Cardiovascular diabetology. 2021;20(1):1-11.
11. Lin Y-H, Lin C-H, Huang Y-Y, Tai A-S, Fu S-C, Chen S-T, et al. Risk factors of first and recurrent genitourinary tract infection in patients with type 2 diabetes treated with SGLT2 inhibitors: a retrospective cohort study. Diabetes Research and Clinical Practice. 2022;186:109816.
12. Tsai M-S, Lin M-H, Lee C-P, Yang Y-H, Chen W-C, Chang G-H, et al. Chang Gung Research Database: A multi-institutional database consisting of original medical records. biomedical journal. 2017;40(5):263-9.

©2024 Pan HC et al. *JAMA Network Open*.
