# Supplementary material for: Sodium-Glucose Cotransport Protein 2 Inhibitors in Patients With Type 2 Diabetes and Acute Kidney Disease
Source: JAMA Netw Open. 2024 Jan 3;7(1):e2350050. doi: 10.1001/jamanetworkopen.2023.50050 (PMC10765268; doi:10.1001/jamanetworkopen.2023.50050)
Supplement: Supplement 2. — Data Sharing Statement [file jamanetwopen-e2350050-s002.pdf]

Data Sharing Statement

Pan. Sodium-Glucose Cotransport Protein 2 Inhibitors in Patients With Type 2 Diabetes and Acute Kidney Disease. *JAMA Netw Open*. Published January 03, 2024.
doi:10.1001/jamanetworkopen.2023.50050

Data
Data available: No

Additional Information
Explanation for why data not available: The datasets used and/or analyzed during the current study are available from the corresponding author on reasonable request. However, we do not have individual patient data.
